# Motion‐related artifacts in structural brain images revealed with independent estimates of in‐scanner head motion

**DOI:** 10.1002/hbm.23397

**Published:** 2016-09-16

**Authors:** Neil K. Savalia, Phillip F. Agres, Micaela Y. Chan, Eric J. Feczko, Kristen M. Kennedy, Gagan S. Wig

**Affiliations:** ^1^ Center for Vital Longevity and School of Behavioral and Brain Sciences University of Texas at Dallas Dallas Texas; ^2^ Department of Behavioral Neuroscience Oregon Health & Science University Portland Oregon; ^3^ Department of Medical Informatics and Clinical Epidemiology Oregon Health & Science University Portland Oregon; ^4^ Department of Psychiatry University of Texas Southwestern Medical Center Dallas Texas

**Keywords:** MRI, head motion, artifact, quality control, cortical thickness, aging

## Abstract

Motion‐contaminated T1‐weighted (T1w) magnetic resonance imaging (MRI) results in misestimates of brain structure. Because conventional T1w scans are not collected with direct measures of head motion, a practical alternative is needed to identify potential motion‐induced bias in measures of brain anatomy. Head movements during functional MRI (fMRI) scanning of 266 healthy adults (20–89 years) were analyzed to reveal stable features of in‐scanner head motion. The magnitude of head motion increased with age and exhibited within‐participant stability across different fMRI scans. fMRI head motion was then related to measurements of both quality control (QC) and brain anatomy derived from a T1w structural image from the same scan session. A procedure was adopted to “flag” individuals exhibiting excessive head movement during fMRI or poor T1w quality rating. The flagging procedure reliably reduced the influence of head motion on estimates of gray matter thickness across the cortical surface. Moreover, T1w images from flagged participants exhibited reduced estimates of gray matter thickness and volume in comparison to age‐ and gender‐matched samples, resulting in inflated effect sizes in the relationships between regional anatomical measures and age. Gray matter thickness differences were noted in numerous regions previously reported to undergo prominent atrophy with age. Recommendations are provided for mitigating this potential confound, and highlight how the procedure may lead to more accurate measurement and comparison of anatomical features. *Hum Brain Mapp 38:472–492, 2017*. © **2016 Wiley Periodicals, Inc.**

## INTRODUCTION

Accurate neuroimaging measurements of brain structure are essential for anatomical characterization, between‐modality image registration, and functional localization. Structural magnetic resonance imaging (MRI) can provide high‐resolution measurements of gray and white matter anatomy that are often the focus of within‐ and between‐participant comparisons of aging [see Dickerson et al., 2009; Fjell et al., 2009; Fotenos et al., 2005], development [e.g., Tamnes et al., 2010], clinical disorders [e.g., Cannon et al., 2015; Dickerson et al., 2009; Kempton et al., 2011], and therapeutic intervention [e.g., Bearden et al., 2008; Dazzan et al., 2005]. In practice, structural MRI scans are readily analyzed with convenient, automated image segmentation tools that derive measurements from an individual's regional neuroanatomy (e.g., thickness, surface area, volume), often implemented with freely available software packages [e.g., FreeSurfer [FS], VBM8, FSL‐VBM; Ashburner and Friston, 2000; Dale et al., 1999; Fischl et al., 1999a; Smith et al., 2004] that have been externally validated with manual tracing and post‐mortem analyses [Cardinale et al., 2014; Kennedy et al., 2009; Kuperberg et al., 2003; Rosas et al., 2002; Salat et al., 2004; Sanchez‐Benavides et al., 2010].

A combination of objective precision and ease of rapid quantification makes the automatic measurement of anatomy a practical method for studying brain morphometry in healthy and diseased populations. The performance of many segmentation algorithms relies on features of image intensity, probabilistic matching to tissue‐type priors, and local spatial relationships between expected brain structures. Consequently, the accuracy of measures extracted from structural MRI is largely contingent on initial image quality, which is sensitive to multiple sources of variability. For example, differences at the participant level [e.g., gray and white matter intensity contrast; Westlye et al., 2009], and instrument‐related noise [e.g., image gradient distortions; Jovicich et al., 2006] may both significantly influence estimates of brain structure [also see Gronenschild et al., 2012; Han et al., 2006].

In line with the above, in‐scanner head motion during MRI has been observed to induce structured and often visually detectable artifacts in brain images [e.g., ringing, blurring; Bellon et al., 1986; Wood and Henkelman, 1985; Zaitsev et al., 2015]. Substantial emphasis has been placed on characterizing how motion‐induced artifacts affect echo‐planar imaging (EPI): both in functional MRI [fMRI; Power et al., 2014; Satterthwaite et al., 2012; Siegel et al., 2014; Van Dijk et al., 2012; Zeng et al., 2014] and diffusion weighted imaging [DWI; Koldewyn et al., 2014; Thomas et al., 2014; Yendiki et al., 2013]. There has been less focus on characterizing how spurious motion‐related biases impact high‐resolution T1‐weighted (T1w) images. This has been due, in part, to limitations in acquiring direct estimates of head motion during T1w sequences. A recent study showed that measures of brain structure from T1w scans contaminated by experimentally induced motion were reliably different from uncontaminated scans of the same individuals [Reuter et al., 2015]. Specifically, instructed patterns of head motion during structural MRI resulted in underestimates of gray matter volume and thickness in healthy young adults. While that report did not detail how the degree of instructed movements related to the natural variation in the types and magnitudes of motion observed across individuals, many cross‐cohort studies contrast individuals who are likely to differ in their degree of motion during MRI [e.g., older vs. younger subjects, children with autism spectrum disorders vs. healthy controls; Chan et al., 2014; Koldewyn et al., 2014; Yendiki et al., 2013]. As a result, the variability in participant motion could systematically bias analyses of structural differences [e.g., Alexander‐Bloch et al., 2016].

Without employing procedures that prospectively correct or remove head motion‐induced artifacts from anatomical scans [e.g., PROPELLER, PROMO, volumetric navigators, Pipe, 1999; Tisdall et al., 2012, 2016; White et al., 2010], biases due to variability in head motion are likely to confound studies of brain structure in addition to any processing steps or statistical analyses that rely on accurate measurements of brain anatomy (e.g., localization of functional activations, surface‐mapping, registration of functional and anatomical images between participants). Accordingly, until quantification and correction tools are further developed and sufficiently adopted for T1w MRI, a practical alternative is necessary in order to advance the interpretation of anatomical measurements. One method for identifying scans with potential motion contamination is to visually inspect structural scans for artifacts and screen them out [as done in Reuter et al., 2015]. While this visual inspection technique is commonplace in structural neuroimaging, it has inherent limitations intrinsic to many subjective procedures including the presupposition that all forms of motion‐related bias are detectable by visual inspection and the possibility that subjective quality assessments may exhibit high inter‐ and within‐rater variability [e.g., Mantyla et al., 1997; Scheltens et al., 1997]. To overcome some of these preceding limitations, we propose some intuitive hypotheses: (1) participants who move more in one scan of a given scan session will move more in other scans collected during the same session, (2) scans that acquire movement estimates may be used to flag structural scans that lack direct estimates of motion but contain motion‐induced artifacts, (3) many of the anatomical scans flagged by high movement may not otherwise be identified by visual inspection alone, (4) flagging potentially problematic scans can help mitigate the effects of movement on brain morphometry, (5) the anatomical scans flagged for movement and poor experimenter‐defined image quality will exhibit systematically biased estimates of brain structure, and (6) removing flagged scans from an estimation sample will influence the measurement of brain structure.

How might we begin to test these predictions? In addition to T1w structural data, many study designs acquire functional scans (e.g., task‐evoked, resting‐state) for which frame‐to‐frame motion estimates are routinely derived. The primary objective of this report was to determine whether the measurements of head motion quantified during these functional scans (e.g., frame‐by‐frame displacements [ FD]) might benefit the identification of structural brain scans that contain motion‐related bias. Specifically, we predicted that FD accurately summarizes individual differences in scanner motion such that one's relative rank within the distribution of average FD values is consistent across fMRI scans. We hypothesized that this relationship would extend to T1w acquisitions whereby increasing FD would be associated with reduced QC ratings of T1w scan quality. Lastly, we predicted that elevated average FD and low QC ratings could be combined to flag subsets of participants whose T1w structural scans are most likely susceptible to motion‐related bias. In addition to determining whether the movement‐related features noted above exist, we intended to measure the impact of motion‐related bias on the measurement accuracy of anatomical differences (e.g., regional thickness) detected over the healthy adult lifespan.

We analyzed data from 266 healthy adult participants, age 20–89, in order to (1) examine the correlation of individuals' tendency to move during scans where head motion is currently measureable (e.g., fMRI), (2) test how well the motion estimates from these independent scans complement the subjective quality control (QC) ratings of T1w anatomical scan quality, and (3) determine whether removing scans “flagged” by a combination of QC ratings and consistently elevated FD alters the measured effects of both aging and motion on brain morphometry. The current dataset allowed extensive measurement of head motion across several EPI scans with differing task‐demands in a single session using the same scanner and scanning protocol. Furthermore, alongside careful estimation of each individual's anatomy (e.g., semi‐automated FS processing), the dataset provided a well‐balanced sampling of the healthy adult lifespan (e.g., at least 30 individuals in each decade of age between 20 and 90 years) with substantial variance in average magnitude of head motion for quantifying individual differences. Crucially, given the purposes of this investigation, accurate quantification of individual variability in movement and anatomy could be considered jointly in an extensive dataset where age‐related observations could be systematically assessed.

## METHODS

### Participants

**Figure 1:**
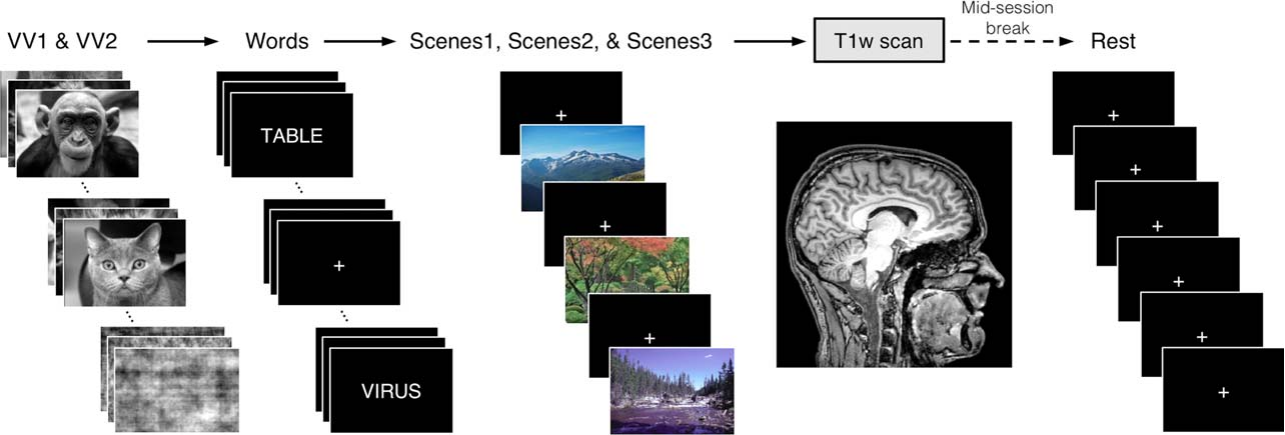
MRI scanning protocol. The Dallas Lifespan Brain Study data acquisition protocol included a T1w anatomical (T1) scan and seven functional MRI (BOLD) scans. Healthy adult participants (*n* = 266, 20–89 years) were each imaged in a single session on the same MRI scanner. Acquiring seven fMRI runs allowed thorough examination of the variation in average head movement (i.e., frame‐by‐frame displacements [ FD]) within and between individuals (see text and Table I for scan information). Conversely, T1w quality control (QC) was performed using researcher‐defined quality ratings. While all data were collected in a single imaging session, participants took a brief mid‐session break, (i.e., 10–15 min) exiting the scanner following the T1w acquisition. Of the BOLD scans, “VV” and “words” employed block design whereas “scenes” and “rest” were event‐related designs. “Words” and “scenes” tasks involved active behavioral responses in response to stimulus delivery (i.e., button‐press), while “VV” and “rest” scans involved passive viewing of visually presented stimuli. Combining estimates of EPI head motion from various fMRI scans with T1w QC ratings allowed close inspection of potential motion‐related bias in estimates of anatomy. [Color figure can be viewed at http://wileyonlinelibrary.com.]

The present sample is a subset of healthy adult participants (*n* = 266) aged 20–89 years (*M* = 54.5, *SD* = 20.4, 169 female) enrolled in the Dallas Lifespan Brain Study (DLBS). This subsample includes at least 30 participants in each decade of the sampled age range and represents individuals who performed the complete series of seven fMRI runs [e.g., Chan et al., 2014; Kennedy et al., 2015; Park et al., 2012, 2013; see Fig. 1]. Critically, this inclusion criterion allowed extensive measurement of participant in‐scanner head motion across a variety of task categories with differing behavioral demands (e.g., scans including “active” behavioral response demands versus scans requiring no overt behavioral responses).

**Table 1:** MRI scan information

Run name	*N*	Acquisition	Frames collected	Task type
*VV1*	266	BOLD	202	Passive
*VV2*	266	BOLD	202	Passive
*Words*	266	BOLD	231	Active/Button‐press
*Scenes1*	266	BOLD	171	Active/Button‐press
*Scenes2*	265	BOLD	171	Active/Button‐press
*Scenes3*	266	BOLD	171	Active/Button‐press
*T1‐weighted* *	266	MPRAGE	1	N/A
*Rest*	266	BOLD	154	Passive

*T1‐weighted scan is followed by a mid‐session break where participants exit scanner.

Participants were recruited from the Dallas‐Fort Worth community and provided written consent before participation. All study procedures were reviewed and approved by the Institutional Review Boards of The University of Texas at Dallas and The University of Texas Southwestern Medical Center. All participants were native English‐speaking and right‐handed with no self‐reported history of neurological or psychiatric disorders. Participants with Mini‐Mental State Examination (MMSE) scores below 26, a history of chemotherapy in the past five years, a coronary bypass, major substance abuse, disorders of the immune system, loss of consciousness for more than 10 minutes, or any MRI safety contraindications were excluded during the recruitment phase. All participants had normal or corrected‐to‐normal visual acuity of 20/30 or better on a Snellen eye chart.

### Experimental Design and MR Acquisition

The DLBS includes data collected with a number of imaging modalities and extensive cognitive and neuropsychological testing across a large age range of individuals. During the MRI session, a T1w structural MRI scan and seven fMRI scans were collected using a Philips Achieva 3.0T scanner (Table 1). In short, this involved: a sagittal magnetization‐prepared rapid gradient echo (MPRAGE) three‐dimensional T1w anatomical scan (TR = 8.1 ms, TE = 3.7 ms, TI = 1,100 ms, flip‐angle = 12°, shot interval = 2,100 ms, FOV = 204 × 256 mm, 160 sagittal slices with 1 mm^3^ voxels, and scan duration = 3 min and 57 s), and seven Blood Oxygenation Level Dependent (BOLD) acquisitions (all functional runs: TR = 2000 ms, TE = 25 ms, flip‐angle = 80°, FOV = 220 mm × 220 mm, 43 interleaved axial slices per volume, 3.5/0 mm (slice‐thickness/gap) in‐plane resolution = 3.4 × 3.4 mm).

The MRI protocol is depicted in Figure 1. The seven BOLD runs comprised four task categories (“VV,” “words,” “scenes,” and “rest”; Table 1) collected in this order: two runs of a passive viewing ventral‐visual stream localizer task (“VV1” and “VV2” scans; 202 frames each), a semantic classification task (e.g., “living”/“nonliving” judgment; “words” scan; 231 frames), three runs of an incidental encoding task (e.g., “water”/“no water” judgment of outdoor scenes; “scenes1,” “scenes2,” and “scenes3” scans; 171 frames each), and a single resting‐state scan (“rest” scan; 154 frames). The T1w scan was collected immediately after the third run of the incidental encoding task (scenes3) for all participants. Following the T1w acquisition, participants exited the magnet for a short break before returning for their resting‐state fMRI scans. One participant did not provide complete data for their scenes2 scan (see Table 1), but was retained in the sample as they contributed sufficient functional and structural data to estimate the effects presented in this report. Experimenters verified that participants complied with all scan instructions via verbal confirmation (see Supporting Information for extended fMRI task instructions).

### Data Preprocessing

#### fMRI preprocessing

BOLD images were preprocessed to reduce known artifacts. Five “dummy” volumes were first discarded from the beginning of each functional run to allow the MR signal to reach steady state. Pre‐processing involved: (i) correction of slice intensity differences attributable to the interleaved acquisition within each TR, and (ii) motion correction for head movement within and across runs. Motion correction was performed with SPM8's realignment procedure, which applies a least squares approach to perform a six‐parameter (three translational and three rotational) rigid‐body transformation for every functional frame to a reference image [Friston et al., 1995]. Realignment was performed within‐participants for each run by estimating the transformation matrix of every functional frame relative to the very first volume collected in the applicable task category (e.g., all frames from VV1 and VV2 scans were realigned to the very first frame of the VV1 run, whereas every frame of the words scan was realigned to the first frame in the words scan).

#### Head motion estimates

In‐scanner head motion for each functional run was quantified with frame‐by‐frame displacement ( FD) as in Power et al. [2012]. In short, for a given fMRI run the six realignment parameters estimated from SPM8 (three translations in mm and three rotations in radians) indexed the absolute displacement of the participant's head at each TR relative to the first EPI frame collected for the task. First, rotational estimates were converted from radians to mm displacements relative to a sphere with a radius of 50 mm (approximate distance from cerebral cortex to center of head). The resulting six‐dimensional time‐series was differentiated in order to index the relative displacement of the participant's head in each dimension for each EPI frame relative to its immediately preceding frame across the task. FD was calculated as the sum of the absolute values of the six differentiated realignment parameters at each frame of a scan (i.e., total movement for each frame was summarized as a positive displacement value).

Average frame‐by‐frame displacements ( $\bar{FD}$) were calculated for each individual within each of their task scans and are distinguished by subscripts throughout this report (Fig. 1). Task $\bar{FD}$, which indexed the amount of motion occurring across a given task, was calculated for each participant as the average of the their $\bar{FD}s$ across individual runs of the same task (e.g., ${\bar{FD}}_{VV}$ was the average of ${\bar{FD}}_{VV1}$ and ${\bar{FD}}_{VV2}$). Task $\bar{FD}s$ were further collapsed into ${\bar{FD}}_{all-task}$ (average $\bar{FD}$ across all four task types). Averaging independent task $\bar{FD}s$ also minimized possible biasing as a function of the number frames collected in a given run or the number of runs contributing to a given task.

#### T1‐weighted image quality control (QC) ratings

All T1w scans were visually checked and rated for scan quality by two researchers (N.S. and P.A.). Raters evaluated images for both motion‐related artifacts [e.g., ringing, blurring, ghosting, and striping; Bellon et al., 1986; Wood and Henkelman, 1985; Zaitsev et al., 2015] and artifacts related to other general properties of brain image quality, such as head coverage, radiofrequency noise, gradient distortions, signal inhomogeneity, wrapping, and susceptibility artifacts [Bennett et al., 1996; Ericsson et al., 1988; Pusey et al., 1986; Sled and Pike, 1998; Vargas et al., 2009]. As in Reuter et al., raters attributed an overall qualitative quality control (QC) assessment of scan quality to each individual's structural image on a three‐category scale (“pass,” “warn,” or “fail”) according, in part, to previously documented criteria [Reuter et al., 2015, http://cbs.fas.harvard.edu/science/core-facilities/neuroimaging/information-investigators/qc]. We include a description of the criteria we used in the supplementary section of this report and also provide extensive documentation so that other researchers may adopt similar screening procedures (see below and Supporting Information).

#### Morphometric estimates

Estimates of brain morphometry were quantified with the default image‐processing pipeline of FreeSurfer v5.3 [FS; Dale et al., 1999; Fischl et al., 1999a], which provided volumetric segmentation and surface‐based cortical reconstruction of individuals' anatomical data. This involved brain extraction using a hybrid watershed/surface deformation procedure, volumetric segmentation, tessellation‐based generation of white matter (WM), and pial surfaces, inflation of the surfaces to a sphere, and surface shape‐based spherical registration of each individual's “native” surface renderings to the FsAverage atlas [Dale and Sereno, 1993; Fischl et al., 1999b, 2004; Segonne et al., 2004, 2005].

Whereas segmentation provided general measures of volumetric anatomy (e.g., gray matter [GM] volume, white matter [WM] volume, subcortical GM volume), surface reconstructions allowed estimates of GM thickness and surface area. Surface area was calculated as the sum of tessellated areas at each location (i.e., vertex) over the full cortical GM‐WM boundary in an individual's “native” surface representation. Cortical GM thickness was calculated as the distance between the GM‐WM boundary (“white” surface) and the outer cortical surface (“pial” surface) at each point across the cortical mantle. This surface‐based thickness estimation is not restricted to voxel resolution of the original T1w image and can detect sub‐millimeter differences between groups [Fischl and Dale, 2000] validated with both histology and manual tracing [Rosas et al., 2002; Kuperberg et al., 2003].

FS processing has been demonstrated to have high test–retest reliability in identifying and measuring various aspects of brain anatomy across scanner manufacturers and field strengths [Dickerson et al., 2008; Han et al., 2006; Jovicich et al., 2006; Morey et al., 2010; Reuter et al., 2012]. However, it is important to point out that the FS processing pipeline requires careful inspection of processed outputs to ensure that segmentations and reconstructions are spatially accurate and anatomically correct. At times, manual intervention is required to correct errors related to inaccuracies in the Talairach atlas transformation, insufficient removal of non‐brain tissue (e.g., dura mater along superior aspects of cortex), inclusions of vessels or other tissue that neighbor the cortex (e.g., often near temporal, orbitofrontal or posterior occipital locations), and field inhomogeneities or inadequate intensity normalization that obscure the GM‐WM boundary. In datasets of aging and/or clinical populations, manual intervention is particularly important because of possible true anatomical abnormalities (e.g., atrophy‐induced uncertainty in tissue‐type boundaries, white matter hyper‐intensities imaged with fluid‐attenuated inversion recovery, enlarged ventricles) that may be resolved inappropriately by the default FS pipeline.

Through an iterative process, FS data for each individual included in this report was visually inspected, edited for inaccuracies (with re‐checking and re‐editing, as needed), and verified by an independent researcher. All researchers involved in editing were instructed from the official FreeSurfer Wiki and editing tutorials (http://freesurfer.net/fswiki/FreeSurferWiki; https://surfer.nmr.mgh.harvard.edu/fswiki/FreeSurferBeginnersGuide), an in‐house guide to our laboratory's FS editing procedures, and in‐person training sessions with a more experienced researcher. We have posted an up‐to‐date “live” manual of in‐house FreeSurfer processing procedures to our laboratory webpage (http://vitallongevity.utdallas.edu/cnl/publications) so that other researchers may follow the recommendations and editing procedures we highlight in the manuscript, and as they are continually refined. Details of the editing procedure and instructions are also included in the supplementary section of this report (Supporting Information). Particular attention was paid in fixing poor skull removal, correcting improperly segmented WM, applying control points to improve tissue classification near the GM‐WM boundary, and ameliorating persistent defects in surface reconstruction.

In addition to the manual editing noted above, each participant's intracranial volume (ICV) was manually traced [see Kennedy et al., 2009] and used to correct for all statistical effects related to volumetric measures of brain structure. A number of studies have suggested that head size (e.g., ICV) covaries with gray and white matter volume and cortical surface area, but generally not with GM thickness [Im et al., 2008; Pakkenberg and Gundersen, 1997]; accordingly, participant gender was used instead of ICV as a nuisance variable for statistical analyses of GM thickness [Barnes et al., 2010]. The results of this report remained qualitatively similar when using both covariates in statistical models. Recent evidence also suggests that FS pipeline outputs may differ based on FS version, workstation and operating system [Gronenschild et al., 2012]; all processing in this study was performed using FreeSurfer v5.3 on a single Enterprise Linux (CentOS 6.6) server.

Surface‐based thickness maps were generated by first bringing the fsaverage‐registered left and right hemisphere anatomical surfaces into register with each other using deformation maps from a landmark‐based registration of the left and right fsaverage surfaces to a hybrid left–right fsaverage surface [fs_LR; Van Essen et al., 2012a] and resampled to a resolution of 163,842 vertices per hemisphere (164k fs_LR) using Caret tools [Van Essen et al., 2001]. Each individual's “native” FS‐generated left and right hemisphere surfaces were deformed to the left and right 164k fs_LR surface meshes using single deformation maps, allowing for minimal resampling of anatomical data. Vertex‐wise thickness estimates for each participant were deformed from “native” FS‐derived surfaces to 164k fs_LR space with the same deformation maps used in the original registration. Individuals' cortical thickness maps were smoothed with a 15 mm kernel (full width‐half maximum, [FWHM]) within the surface representations of each of the two hemispheres. This smoothing parameter was adopted so as to be comparable to previous surface‐based reports examining measurements of cortical thickness [e.g., Fjell et al., 2009; Reuter et al., 2015].

### Statistical Analysis

#### Within‐session stability of fMRI head motion

We first tested whether a participant's average head motion ( $\bar{FD}$) was strongly correlated across scans collected within the same session. The associations between all pairs of scan $\bar{FD}$ were quantified using Spearman's rank‐order correlation coefficients (Spearman's rho) because measures of $\bar{FD}$ were significantly non‐normal (skewed right and leptokurtic) and relationships among the $\bar{FD}s$ of various runs exhibited heteroscedasticity (see Supporting Information). However, the results of this report remained qualitatively similar when using Pearson's correlations. The scan $\bar{FD}$ values measured across participants were correlated between each pair of scans using a significance threshold of *P* < 0.05 after Bonferroni correction for 21 simultaneous comparisons. The significance of each pair‐wise correlation (e.g., ${\bar{FD}}_{scan1}\;vs.\;{\bar{FD}}_{scan2}$ across participants) was further confirmed with a permutation test using the following randomization procedure: (1) the vector of ${\;\bar{\;\;FD}}_{scan1}$ values (one value for each participant) measured during one scan was randomly reordered without replacement resulting in ${\bar{FD}}_{{scan1}^{'}}$, (2) the correlation was measured between ${\bar{FD}}_{{scan1}^{'}}$ and the intact (not reordered) vector of ${\bar{FD}}_{scan2}$ values measured during a second scan, and (3) steps 1 and 2 were performed 100,000 times to generate a null distribution of correlation coefficients for comparison with the actual measured value.

#### Relationship of fMRI head motion and visually‐detected T1w artifacts

To examine if increasing fMRI head motion could be used to detect increasing T1w artifacts, we tested whether ${\bar{FD}}_{all-task}$ differed systematically across the categories of T1w image quality ratings. We computed the nonparametric Kruskal–Wallis H‐test of ${\bar{FD}}_{all-task}$ as predicted by QC ratings, where the group‐differences in ${\bar{FD}}_{all-task}$ were further analyzed using Wilcoxon rank‐sum tests with a significance threshold of *P* < 0.05 after Bonferroni correction for three simultaneous comparisons. To determine whether the correspondence of QC ratings and ${\bar{FD}}_{all-task}$ was driven by collinearity with age, we computed an analysis of covariance (ANCOVA) model of the independent effects of participant age (continuous) and QC ratings (categorical) on the dependent variable ${\bar{FD}}_{all-task}$.

#### Distinct variance from fMRI head motion versus QC ratings

To test the overlap in participants flagged by ${\bar{FD}}_{all-task}$ (provisional cutoff of 1.5SD > sample mean ${\bar{FD}}_{all-task}$) versus those flagged by QC ratings we calculated the sensitivity and specificity of the “fail” QC categorization on detecting individuals flagged as having elevated ${\bar{FD}}_{all-task}$. Sensitivity measured the proportion of participants flagged by FDall‐task (i.e., ${\bar{FD}}_{all-task}$ greater than 1.5SD above the sample mean) that were correctly identified by quality ratings of “fail,” whereas specificity quantified the proportion of participants not flagged by FDall‐task (i.e., ${\bar{FD}}_{all-task}$ less than 1.5SD above sample mean) correctly identified with quality ratings of either “pass” or “warn.” This analysis was complemented with a permutation test to quantify the likelihood that the actual measured values of sensitivity and specificity were due to chance alone (i.e., significance): 100,000 groups of 17 participants (number of QC “fails”) were randomly resampled without replacement from the full set of 266 participants, from which null sensitivity and specificity distributions were calculated by comparing each resampled group of 17 against the 18 high‐ ${\bar{FD}}_{all-task}$ participants.

We hypothesized that ${\bar{FD}}_{all-task}$ and QC ratings would predict independent variance in FreeSurfer‐derived estimates of GM thickness. To test this hypothesis, two ANCOVA models were constructed to compare ${\bar{FD}}_{all-task}$ and QC ratings to one another and to morphometry derived from FreeSurfer. The first ANCOVA model calculated the variation in GM thickness estimates (dependent variable) predicted by the independent effects of participant age (continuous), ${\bar{FD}}_{all-task}$ (continuous), and QC ratings (categorical) with gender (categorical) used as a covariate. The effect size (partial eta‐squared) of age on thickness was calculated before and after controlling for ${\bar{FD}}_{all-task}$ and QC ratings; to determine if the change in effect size was greater than that expected by chance, the actual difference in effect size was compared with a null distribution of differences in effect sizes derived by permuting ${\bar{FD}}_{all-task}$ and QC ratings across participants in 1,000 iterations. The above ANCOVA model was calculated twice: once including the full participant sample and once after removing scans flagged by a combination of QC ratings and ${\bar{FD}}_{all-task}$. ANCOVA models were conducted with all interactions terms included (using type III sum of squares); main effects of independent variables were recomputed without controlling for interaction terms if no significant interactions were detected.

#### Reductions in motion‐related bias after flagging

Next, it was crucial to test how our flagging procedure impacted the effect of motion on thickness values measured across the cortical surface. We calculated vertex‐wise full‐partial correlations of thickness and ${\bar{FD}}_{all-task}$ (controlling for age and gender) before and after removing the flagged scans. The vertex‐wise map obtained after removing flagged scans was compared with the 95% confidence interval from 1,000 re‐sampled control groups of the same size as the retained sample (*n* = 235); each control group was built by randomly removing 1 participant from the 10 retained individuals closest in age and of the same gender as each participant in the flagged group. The true shift in the associations between thickness and ${\bar{FD}}_{all-task}$ was considered significant if the cumulative distribution function fell outside that of the estimated 95% confidence interval.

#### Systematically biased morphometry in flagged scans

It was crucial to determine whether those participants suspected of having motion‐related bias in their T1w structural scans (i.e., flagged by either elevated ${\bar{FD}}_{all-task}$ or a QC rating of “fail”) exhibited systematic differences in FreeSurfer‐based thickness relative to demographically similar individuals. We performed a bootstrap resampling analyses to create 100,000 age‐ and gender‐matched control samples and compared the resulting distribution of mean GM thickness values with that of the flagged group. Each control sample was generated by randomly selecting (with replacement) one participant from the ten retained individuals closest in age and of the same gender as each member of the flagged group. We then calculated the probability of measuring the observed group mean thickness for the flagged participants relative to the null distributions of 100,000 group means built from the resampling procedure. The analysis of GM thickness was followed up with a surface‐based comparison of thickness values for the flagged group against a randomly selected bootstrapped control sample. Vertex‐wise two‐sample *t*‐tests were performed for the two hemispheres independently and controlled for False Discovery Rate (FDR) at a *P* < 0.05 significance threshold.

#### Regional effect sizes of age on morphometry before and after flagging

Lastly, we tested whether removing the 31 flagged scans altered the measured effects of age on average whole‐brain and regional estimates of GM thickness. The variance of average whole‐brain GM thickness and its correlation with participant age were compared both in the full sample (*n* = 266) and after removing the flagged scans (*n* = 235) by Bartlett's test for unequal variances and a *z*‐test for correlation differences, respectively. Additionally, we examined the regional influences of the flagging procedure by calculating two vertex‐wise correlation maps of age and thickness, once before and once after removing flagged participants from the estimation sample. The vertex‐wise correlation maps were first compared by a two‐sample Kolmogorov–Smirnov goodness of fit test to assess whether the overall distribution of effect sizes across the cortical surface had been altered. Then, we contrasted the average correlational effect sizes before and after flagging (*z*‐value difference in Fisher *z*‐transformed *r*‐values) in regions of interest based on the Destrieux anatomical parcellation [Destrieux et al., 2010].

### Computation and Visualization

Several software packages were used in the preparation of data for this manuscript. Motion estimates were derived using Statistical Parametric Mapping (SPM8, Wellcome Trust Center for Neuroimaging, London, United Kingdom), and statistical analyses were performed in R (3.1.3, R Foundation for Statistical Computing, Vienna, Austria) and MATLAB [2013a, The MathWorks, Natick, MA]. Graphical depictions were created using the R‐package, ggplot2 [Ginestet, 2011]. Volumetric images were visualized with FSL tools (Oxford Centre for Functional MRI of the Brain, Oxford, United Kingdom), while surface‐based processing was performed using FreeSurfer [v5.3, Dale et al., 1999; Fischl et al., 1999a] and Connectome Workbench [v0.83, Marcus et al., 2011].

## RESULTS

If the naturally occurring in‐scanner head displacements are determined to be stable for a participant across their scan session, measures of head motion from functional scans, from which movement estimates are obtainable, might provide an objective method for flagging and removing potentially problematic structural data. Accordingly, we first sought to determine whether individual differences in the magnitude of $\bar{FD}$ were stable across a single scan session.

### Participant Rank in Head Motion is Stable Across Scans Within a Session

**Figure 2:**
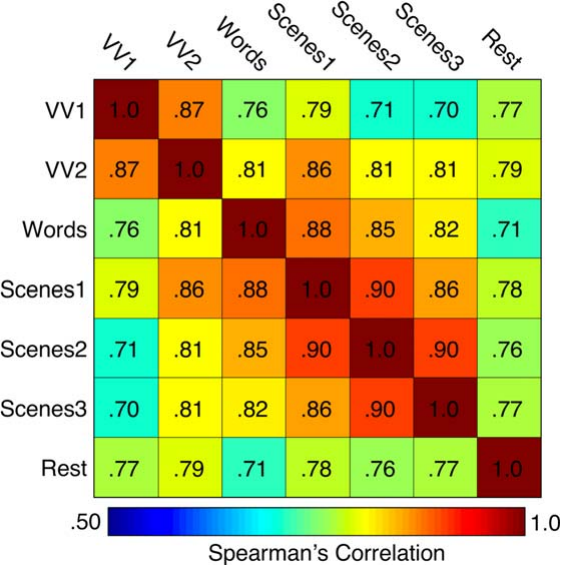
Measures of in‐scanner head motion are stable within individuals across scans. Individual variability in $\bar{FD}$ is highly correlated across each pair of fMRI scans within a scanning session (all *P*s < 0.001 relative to 100,000 permutated control samples after Bonferroni correction for 21 pair‐wise comparisons). Despite the robust scan‐to‐scan relationships, the cross‐correlation matrix appears to exhibit some heterogeneity (*r*'s range from 0.70 to 0.90). [Color figure can be viewed at http://wileyonlinelibrary.com.]

For all seven functional scans, the pair‐wise correlations between all pairs of scan $\bar{FD}$ were very high and positive (all *r*s > 0.70, all *P*s < 0.001 after Bonferonni correction for 21 simultaneous comparisons; Fig. 2) despite a main effect of scan order on magnitude of $\bar{FD}$ (*F*(6, 1,854) = 5.86, *P* < 0.001). Permutation tests confirmed that all measured correlation values were extremely unlikely to be due to chance alone (all *P*s < 0.001), with no randomly resampled control group showing a correlation that exceeded the measured values. Altogether, participants were consistently ranked by their $\bar{FD}$ across the scanning session despite differences in task demands, time‐lags between scans, and even breaks where they exited the scanner (i.e., between scenes3 and rest scans). Given the highly significant within‐participant relationship in run‐to‐run $\bar{FD}$, a participant's average $\bar{FD}$ across all scans may reliably quantify the motion‐related bias expected in that individual's other scans.

### Average Frame‐by‐Frame Displacement Tracks Rater‐Defined T1‐Weighted Image Quality

Given the significant associations between the average head displacements across functional scans, the stability of average fMRI motion estimates may extend to T1w images and be used to infer the presence of problematic T1w structural scans. To test this hypothesis, we first analyzed the inter‐rater reliability and distribution of experimenter‐defined QC ratings of T1w scans, and then compared $\bar{FD}$ to the subjective categorizations.

#### T1‐weighted image quality ratings

Despite the use of standard criteria and considerable experience with rating (see Supporting Information), inter‐rater reliability was moderate (Cohen's *κ* = 0.482, 95% *CI* = 0.367–0.600, *P* < 0.001) with all inter‐rater disagreements occurring for scans given QC ratings of adjacent categories (i.e., no instances where one rater marked a scan as “fail” when rated “pass” by the other). Table 2 indicates that raters had difficulty in dissociating levels of poorer T1w quality (41.2% overlap in “fail” ratings, 38.7% overlap in “warn” ratings), whereas comparatively better consistency was observed for scans rated “pass” (75.9% overlap). We emphasize that the sizable cross‐rater variability suggests that the range of possible T1w artifacts may not be well represented by the category distinctions of subjective raters and reinforces the motivation to identify more quantitative approaches.

**Table 2:** T1‐weighted image QC ratings

QC ratings	*“Pass”*	*“Warn”*	*“Fail”*	
Generalized assessment criteria	“Noise/artifacts are either undetectable or faintly detectable; overall visual image quality unaffected”	“Moderate spatially‐contained noise/artifacts present in multiple image slices; overall visual image quality mildly affected”	“Severe noise/artifacts pervasive, present throughout majority of image; resulting data may be unusable”	
*N* (Rater 1)	187	71	8	
*N* (Rater 2)	186	64	16	
% Overlap	75.9%	37.8%	41.2%	
*N* (Composite)	161	88	17	
Age range (Mean, *SD*)	20–88 years (*M = *48.2, *SD = *18.9)	22–89 years (*M = *62.4, *SD = *19.3)	30–86 years (*M* = 72.7, *SD* = 14.0)	
% of total sample	60.53%	33.08%	6.39%	
*N*_YA_ (Total = 64)	52	11	1	*X* ^2^ versus expected counts based on *N*_YA_ *
*N*_ME_ (Total = 53)	39	14	0	*X* ^2^(2) = 2.20, *P* = 0.332
*N*_ML_ (Total = 52)	34	15	3	*X* ^2^(2) = 4.19, *P* = 0.123
*N*_OA_ (Total = 97)	36	48	13	*X* ^2^(2) = 30.93, *P* < 0.001

*Each *X* ^2^ test was performed using an expected proportion of ratings based on the YA cohort. The sample sizes of ME and ML differ substantially from OA, which may accentuate differences in the resulting *X* ^2^ statistic.

*YA, younger adults (20*–*34 years); ME, middle early adults (35*–*49 years); ML, middle late adults (50*–*64 years); OA, older adults (65*–*89 years)*.

To exercise caution in flagging problematic anatomical data, the lower of the two valuations (more stringent) from the raters determined the final category label given to each scan (e.g., one rating of “warn” and another rating of “pass” resulted in a final label of “warn”). Ultimately, 161 T1w scans were labeled “pass” (60.5%), 88 labeled “warn” (33.1%), and 17 labeled “fail” (6.4%). This distribution revealed that while the majority of scans were labeled as relatively reasonable quality, a non‐trivial portion (6.4%) was flagged for potential problems. Critically, the majority of the “fail” scans here derive from participants in later adulthood (16/17 from participants over 50 years of age; see Table 2), highlighting a potentially age‐related bias in T1w ratings. It is worth noting that the distribution of image ratings for the young adults in this study (20–34 years of age) does not differ statistically from the distribution of ratings for young adults from Reuter et al. [2015; i.e., relative to the study's “still” condition]. However, the existence of cohort‐based differences in the distribution of QC ratings (e.g., statistical difference in the rating distributions of younger and older adults; Table 2) might render our analysis of anatomical differences between individuals of different ages susceptible to misestimation, a point we return to in a subsequent section.

#### T1‐weighted image quality ratings and EPI head motion

**Figure 3:**
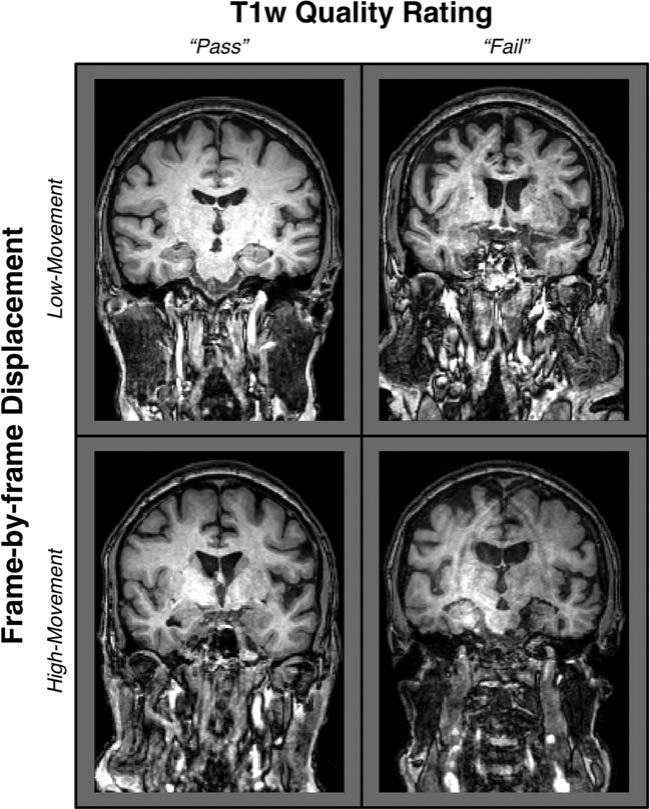
Anatomical scans qualitatively reveal shared and unshared variation between quality ratings and EPI head motion. T1w images drawn from older adults (over 70 years of age) are arranged in a 2 × 2 matrix to highlight that quality ratings and $\bar{FD}$ are related, but provide non‐overlapping characterizations of motion‐related artifacts. An excess of salient rater‐detected artifacts results in a T1w quality assessment of “fail,” which visibly distinguishes the two T1w images on the right side of this diagram from the “pass” images on the left. On the other hand, when artifacts are not subjectively detected or are faintly present (i.e., “pass”), it is unknown whether the T1w scans of a participant with a tendency to move (1.5SD > the group average ${\bar{FD}}_{all-task}$) during fMRI contains more motion‐related bias than that of a person who is less prone to head motion (compare high‐movement “pass” vs. low‐movement “pass”). Importantly, however, it is clear that an increased tendency to move exacerbates image contamination when artifacts are visually detectable (compare high‐movement “fail” vs. low‐movement “fail”). A primary concern is that structural images from high‐motion participants could contain substantial motion‐related bias but be retained in a sample when quality ratings are used without other considerations.

**Figure 4:**
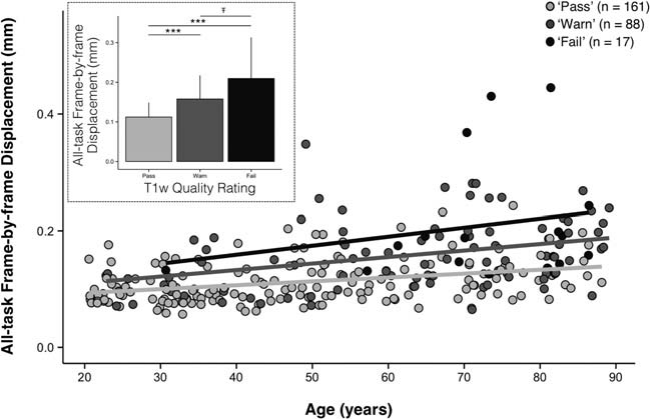
Greater EPI head motion is associated with poorer visual quality ratings independent of age. Increasing age is associated with increasing average head motion ( $\bar{FD}$) over the healthy adult lifespan (*r* = 0.44, *P* < 0.001). Data points are color‐coded by the corresponding rater‐defined quality score in the scatterplot to illustrate that that decreasing T1w image quality ratings correlate with individuals' tendency to move during fMRI. For depiction purposes, separate least‐squares regression lines are shown on the scatterplot for the individual quality categorizations to illustrate the age‐invariant main effect of QC ratings on ${\bar{FD}}_{all-task}$. The accompanying bar plot (inset) verifies that for each descending level of quality, average EPI head motion is significantly greater. *Significance levels for Wilcoxon rank‐sum tests*: Ŧ for uncorrected P < 0.05; *** for Bonferroni corrected P < 0.001.

We hypothesized that individuals with higher $\bar{FD}$ during fMRI scans tend to have greater motion‐induced artifacts in their T1w images (Fig. 3). A Kruskal–Wallis H‐test confirmed that increased ${\bar{FD}}_{all-task}$ was significantly associated with poorer T1w scan quality: *X* ^2^(2) = 57.41, *P* < 0.001 (median “pass” FDall‐task = 0.10 mm, median “warn” FDall‐task = 0.15 mm, median “fail” FDall‐task = 0.17 mm). Pair‐wise Wilcoxon rank‐sum tests (Bonferroni corrected for three simultaneous comparisons; see Fig. 4, bar plot) indicated that scans rated as “pass” were associated with significantly lower ${\bar{FD}}_{all-task}$ than scans labeled “warn” (*U* = 3667, *P* < 0.001, *z* = −6.29, *η* ^2^ = 0.16) or “fail” (*U* = 302, *P* < 0.001, *z* = −5.28, *η* ^2^ = 0.16). Along these lines, scans labeled as “fail” were associated with nominally higher ${\bar{FD}}_{all-task}$ than those labeled “warn” (*U* = 510, *P* = 0.117, *z* = −2.07, *η* ^2^ = 0.04).

Despite the strong relationship between poorer quality ratings and increasing ${\bar{FD}}_{all-task}$, it remained possible that the relationship between these two variables was confounded by a mutual relationship with age. Consistent with this possibility, older age groups exhibited a larger proportion of poorer quality ratings (see Table 2) and increasing age significantly correlated with increasing ${\bar{FD}}_{all-task}$ (*r* = 0.44, *P* < 0.001; Fig. 4). An ANCOVA of ${\bar{FD}}_{all-task}$ (dependent variable) was computed including QC rating as a between‐subject factor and participant age as a continuous predictor (*F*(5, 260) = 24.95, *P* < 0.001, adj. *R* ^2^ = 0.311). With no detectable interaction between quality and age (*F*(2, 260) = 1.21, *P* = 0.301), we recomputed the main effects of QC ratings and age on ${\bar{FD}}_{all-task}$ without controlling for interactions. The main effect of rater‐defined quality on ${\bar{FD}}_{all-task}$ was significant (*F*(2, 260) = 23.23, *P* < 0.001) independent of a main effect of age (*F*(1, 260) = 28.26, *P* < 0.001).

### Elevated $\bar{FD}$ and Poor QC Ratings Predict Independent Variance in Potential Motion‐Related Bias

Although T1w quality assessments and ${\bar{FD}}_{all-task}$ covary, these two measures may capture non‐overlapping aspects of the motion‐related bias present in T1w data. This would not be entirely surprising: the QC ratings assess the visual severity of the artifacts but are inherently subjective, while average ***FD*** from fMRI provides an independent (indirect) measure of one's tendency to move during a T1w scan. To address this question, we tested the overlap in participants given a QC rating of “fail” and those identified as high movers according to ${\bar{FD}}_{all-task}$.

#### $\bar{FD}$ *and QC ratings flag distinct sub‐samples*

**Figure 5:**
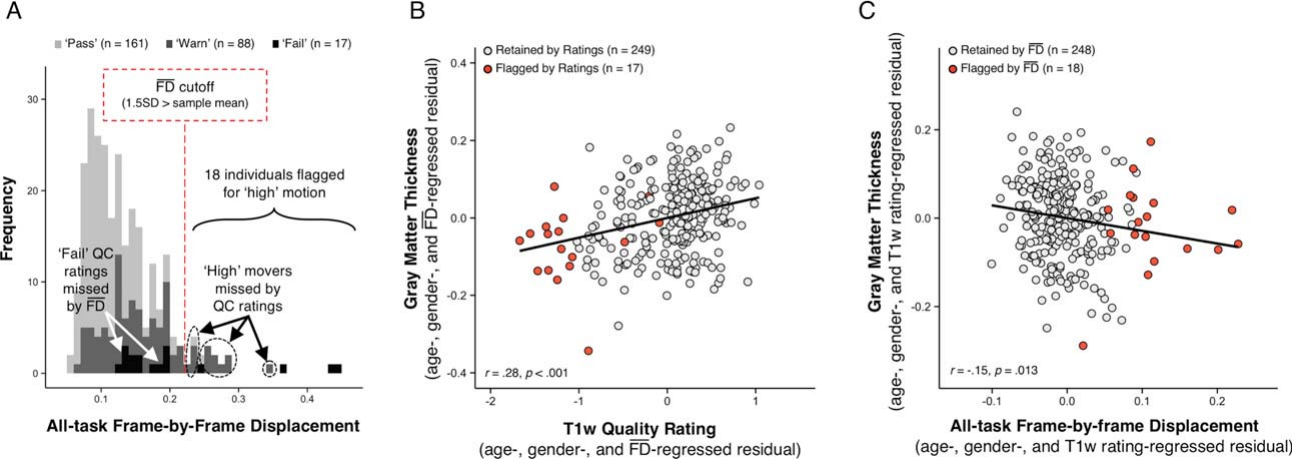
Quality ratings and EPI head motion contribute partially independent sources of potential bias‐related variance. (a) A histogram of ${\bar{FD}}_{all-task}$ illustrates that visual quality ratings miss over 75% of high‐movement participants (low sensitivity). Since flagging participants with elevated ${\bar{FD}}_{all-task}$ (in the present case 1.5SD above sample mean, depicted by red dotted line) similarly misses a number of “fail” images, these two methods of data screening likely need to be considered together to better control for potential motion‐related bias. (b) Lower (poorer) visual quality ratings are significantly associated with decreased thickness estimates after controlling for participant age, gender and ${\bar{FD}}_{all-task}$. Data points that are flagged by a quality rating of “fail” are colored orange to highlight that the lowest T1w quality ratings result in decreased thickness estimates independent of regressed variables. (c) Increasing ${\bar{FD}}_{all-task}$ is significantly associated with reduced estimates of thickness after controlling for age, gender, and quality ratings. High movers flagged by EPI head motion (i.e., 1.5SD above sample mean) are highlighted in orange to show that the highest movers tend to have the lowest thickness estimates relative to their age, gender, and T1w quality. [Color figure can be viewed at http://wileyonlinelibrary.com.]

Participants with ${\bar{FD}}_{all-task}$ greater than 1.5SD above the sample mean were flagged as “higher” motion outliers, identifying the most non‐compliant participants in terms of fMRI head movement (*n* = 18). A sensitivity‐specificity analysis was performed on the capacity for visual quality assessments to identify individuals characterized as being higher movers according to $\bar{FD}$ (Fig. 5A). Although the resulting specificity (0.95) appeared numerically high, sensitivity (0.22) appeared very low. The results of permutation testing suggested that the measured sensitivity was statistically significant (*P* = 0.018), although the specificity failed to reach significance (*P* = 0.065). It is critical to point out that although this result indicates that T1w quality ratings identify scans from high movers better than chance (e.g., sensitivity), data screening with ratings alone would fail to flag over 75% of high‐ $\bar{FD}$ participants in the final sample. Conversely, the majority of participants in the “fail” quality category would not be flagged solely on the basis of having elevated ${\bar{FD}}_{all-task}$. These observations highlight and reinforce the need to use both measures to adequately flag the dataset for possible motion‐related bias.

### Flagging Participants with Elevated $\bar{FD}$ and Poor QC Ratings Reveals Biased Estimates of Morphometry

What is the impact of including motion‐contaminated anatomical scans on measurements of brain morphometry? The findings presented thus far suggest that we have two metrics, QC ratings and ${\bar{FD}}_{all-task}$, for identifying potentially problematic T1w scans. Next, we set out to determine whether and how the potential motion‐related bias highlighted in the flagged participants impacts estimates of morphometry derived from the T1w scans.

#### *Cortical thickness covaries with* $\bar{FD}$ *and QC ratings independent of age*

We constructed an ANCOVA model with QC ratings, ${\bar{FD}}_{all-task}$, and age as predictors of GM thickness (dependent variable) with gender included as a nuisance variable. There were no detectable interactions between the independent variables in this model (all *P*s > 0.501), so the individual main effects of the predictors were assessed without controlling for their interactions. The model fit was significant (*F*(23, 242) = 21.14, *P* < 0.001, adj. *R* ^2^ = 0.636) and there was a significant main effect of increasing age on decreased thickness estimates (*F*(1, 242) = 212.98, *P* < 0.001). In addition, both the main effect of visual QC ratings (*F*(2, 242) = 10.74, *P* < 0.001) and the main effect of ${\bar{FD}}_{all-task}$ (*F*(1, 242) = 4.33, *P* = 0.038) on thickness values were significant, independent of one another, age and gender. Of note, while the effect of age on thickness remained significant after controlling for QC ratings and ${\bar{FD}}_{all-task}$, its effect size (partial eta‐squared) was reduced from 0.59 (without controlling for QC and ${\bar{FD}}_{all-task}$) to 0.47 (significant reduction: *P* = 0.001); we return to this point in a later section. Importantly, the above ANCOVA was re‐calculated after removing scans flagged by QC ratings and ${\bar{FD}}_{all-task}$ (*F*(15, 219) = 23.59, *P* < 0.001, *R* ^2^ = 0.591) and the significant main effects of QC ratings (*F*(2, 219) = 10.66, *P* = 0.001) and ${\bar{FD}}_{all-task}$ (*F*(1, 219) = 6.09, *P* = 0.014) persisted independent of a significant main effect of age on decreasing average whole‐brain thickness (*F*(1, 219) = 190.62, *P* < 0.001).

To better understand the variability in GM thickness estimates predicted by these two measures of motion‐related bias, regression models were constructed by first controlling for the effects of age, gender, and one of the two measures (i.e., partial correlations). In the model controlling for age, gender and ${\bar{FD}}_{all-task}$, decreasing GM thickness was significantly related to poorer QC ratings (*r* = 0.28, *P* < 0.001; Fig. 5B). Conversely, after removing the effects for age, gender and QC ratings, decreasing GM thickness was significantly associated with higher ${\bar{FD}}_{all-task}$ (*r* = −0.15, *P* = 0.013; Fig. 5C). Critically, both of these relationships remain after correcting for multiple comparisons (Bonferroni correction for two tests at significance threshold of *P* = 0.05). We highlight that there is no significant difference in the absolute effect sizes of these two variables on GM thickness (*z* = 1.52, *P* = 0.129), providing additional evidence that both independent measures are important for flagging potentially problematic T1w scans.

#### *Flagging limits the effects of* $\bar{FD}$ *on regional thickness estimates by filtering biased anatomical scans*

**Figure 6:**
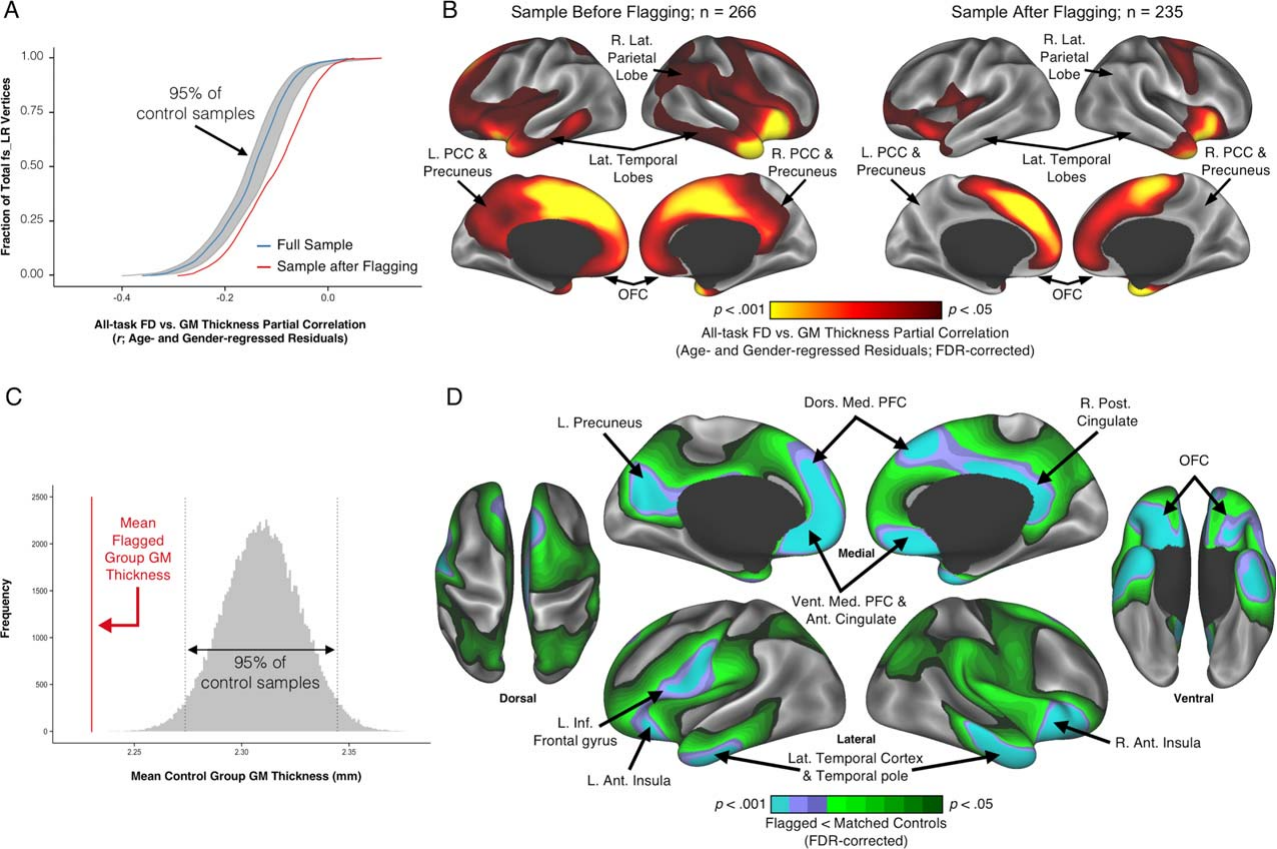
Flagging data using both EPI head motion and QC ratings limits the effects of motion‐related bias in gray matter thickness. (a) The cumulative distribution function of vertex‐wise GM thickness vs. ${\bar{FD}}_{all-task}$ full‐partial correlations (controlling for age and gender) differs before (blue line) and after (red line) removing anatomical scans flagged by a combination of T1w quality ratings (“fail” images) and elevated ${\bar{FD}}_{all-task}$ (1.5SD > sample mean). The vertex‐wise associations of GM thickness versus ${\bar{FD}}_{all-task}$ after removing flagged scans are significantly reduced relative to the 95% confidence interval (gray lines and shaded area) measured from 1,000 randomly re‐sampled control samples (each *n* = 235) from which unflagged scans that are demographically‐matched to the flagged group were removed. (b) Comparing the regional distribution of vertex‐wise GM thickness versus ${\bar{FD}}_{all-task}$ full‐partial correlations (controlling for age and gender; FDR‐corrected) before and after removing flagged scans (left vs. right *P*‐value maps, respectively) indicates that the significant relationships between head motion and thickness are reduced, if not eliminated, across the majority of the cortex. (c) The group of flagged participants (*n* = 31) have average whole‐brain GM thickness estimates significantly lower than bootstrapped age‐ and gender‐matched samples. The global bias highlights that reduced thickness estimates are likely to be consistently derived for flagged images (vertical lines represents 95% confidence interval). (d) A vertex‐wise *t*‐test (FDR‐corrected) between the flagged group and a control sample indicates that the motion‐related bias in thickness estimates is regionally patterned. All the detected differences in this comparison occur in regions that exhibit significantly lower GM thickness for the flagged group (flagged < matched controls). Differences are prominent in association cortex and along the cortical midline, particularly emphasized in brain regions reported to undergo prominent atrophy with age (e.g., dorsal medial PFC, posterior cingulate, temporal poles, OFC, lateral temporal cortex, ventral medial PFC, anterior cingulate, anterior insula, and left inferior frontal gyrus). *L., left; R., right; Ant., anterior; Post., posterior; Inf., inferior; Med., medial; Lat., lateral; Dors., dorsal; Vent., ventral; PFC, prefrontal cortex; OFC, orbitofrontal cortex; PCC, posterior cingulate cortex*. [Color figure can be viewed at http://wileyonlinelibrary.com.]

Given the observed relationships between our measures of interest and average whole‐brain thickness persisted statistically after flagging, it was important to understand the spatial distribution of the effects and whether our flagging procedure may attenuate the potential motion‐related bias regionally. Data from a total of 31 individuals' T1w scans were flagged as potentially problematic for structural estimation (i.e., total combining both rating “fails” and ${\bar{FD}}_{all-task}$ 1.5SD above the sample mean). We calculated the vertex‐wise full‐partial correlations of GM thickness and ${\bar{FD}}_{all-task}$ while controlling for age and gender before and after removing flagged scans from the estimation sample. After removing the 31 flagged scans, the distribution of effect sizes was significantly reduced compared with 1,000 pseudo‐randomly re‐sampled groups of the same size (Fig. 6A). Relative to the full sample, the flagging procedure appeared to reduce if not eliminate the effect of ${\bar{FD}}_{all-task}$ across the vast majority of the cortical surface (compare left and right in Fig. 6B). The regions where the age‐ and gender‐regressed relationships between ${\bar{FD}}_{all-task}$ and GM thickness were eliminated by removing flagged scans notably included the bilateral cingulate cortex, bilateral lateral temporal cortex, and right lateral parietal cortex.

To further characterize the impact of including potentially problematic scans in a study sample we examined the difference between the flagged group and unflagged controls. A bootstrap procedure was used to create gender‐ and age‐matched control samples for the flagged group. The average GM thickness of the flagged group was consistently lower than that of control samples (*P* < 0.001; Fig. 6C), with no control sample having less average thickness than the flagged group. Echoing the results of Reuter et al. [2015], data believed to be susceptible to motion‐related bias resulted in thickness estimates that were lower than could be expected by random variability in the population. As in Reuter et al., measures of gray matter volume also showed a pattern of bias consistent with these primary results (see Supporting Information).

A vertex‐wise two‐sample *t*‐test was used to compare the cortical thickness of the flagged sample and a control group identified in the bootstrapping procedure above (i.e., age‐ and gender‐matched to the flagged sample; see Fig. 6D). Cortical regions exhibiting significantly reduced GM thickness in the flagged sample were distributed across hemispheres and cortical lobes. Bilaterally, these regions included anterior and posterior cingulate, precuneus, anterior insula, dorsal and ventral medial prefrontal cortex (PFC), superior frontal cortex, orbitofrontal cortex (OFC), superior parietal lobule (SPL), lateral temporal cortex, and the temporal poles. Reductions in thickness for the flagged group were also present in the left inferior frontal gyrus (IFG; e.g., pars triangularis, pars opercularis), right supramarginal gyrus (SMG) and right angular gyrus (AG). Consistent with the above finding that thickness was reduced globally in the flagged images, no brain regions in the flagged group were reliably greater than that of the matched control sample.

#### Removing flagged scans attenuates the effects of age on regional thickness estimates

**Figure 7:**
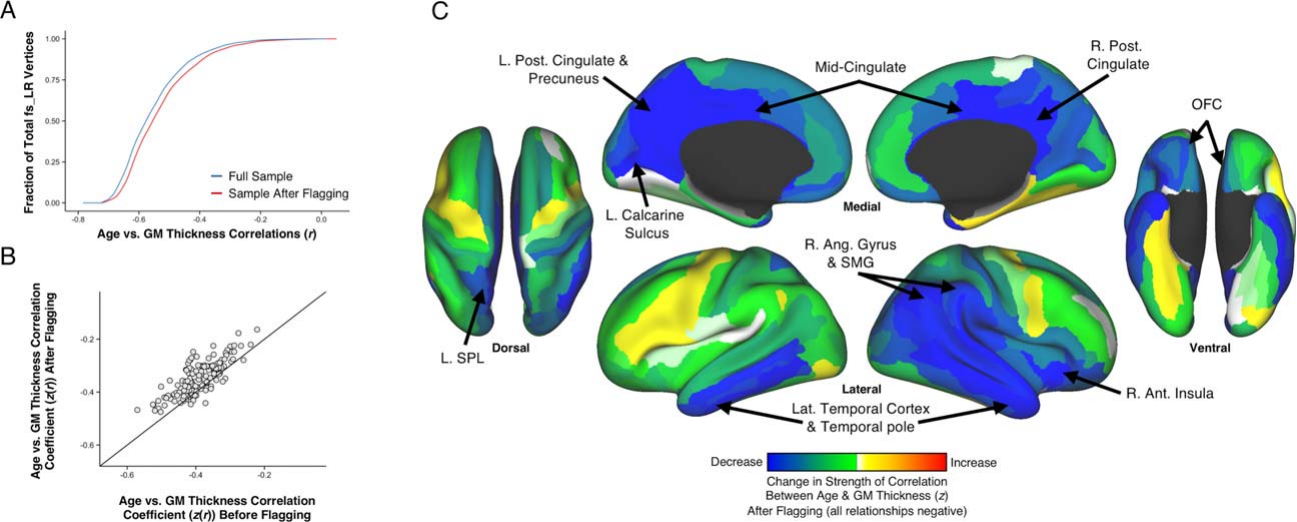
Regional effects of age‐related cortical thinning are reduced when removing flagged scans that are susceptible to potential motion‐related bias. (a) The cumulative distribution function of vertex‐wise age vs. GM thickness correlations differs before (blue line) and after (red line) removing anatomical scans flagged by T1w quality ratings and elevated ${\bar{FD}}_{all-task}$. The subtle rightward shift in correlations (toward *z*(*r*) = 0) indicates a significant reduction in overall effect sizes when structural scans flagged for potentially motion‐related artifacts have been removed from the estimation sample. (b) A scatter plot of the correlations (Fisher's *z*‐transformed *r*‐values) of anatomical parcel thickness versus age before and after removing flagged scans reveals that the majority of regional effect sizes are reduced (86% of parcels) after scans susceptible to potential motion‐related bias have been filtered from the sample (diagonal line indicates no difference). (c) A surface‐based map illustrates that the differences in average correlation between age and GM thickness occur variously across anatomical parcels (all relationships remained negative; color‐scale reflects the strength and direction of correlation change after removing flagged scans). Reduced age effects are prominent along the cortical midline (e.g., cingulate cortex, precuneus, calcarine sulcus), bilateral lateral temporal cortex, right lateral parietal, and right anterior insular cortex, suggesting that these particular regions may be most susceptible to misestimates of age‐related cortical thinning when motion‐related bias is left uncontrolled. *L., left; R., right; Ant., anterior; Ang., angular; Post., posterior; Inf., inferior; Med., medial; Lat., lateral; Dors., dorsal; Vent., ventral; SPL, superior parietal lobule; SMG, supramarginal gyrus; OFC, orbitofrontal cortex*. [Color figure can be viewed at http://wileyonlinelibrary.com.]

Aging is accompanied by regionally specific changes in cortical gray matter. Since age additionally relates to the flagging metrics that are indicative of motion‐related bias it was important to describe how, if at all, flagging altered the relationships between aging and regional thickness estimates. The vertex‐wise correlation between GM thickness and age was measured before and after removing flagged T1w scans. Given that our flagging procedure made a relatively small perturbation to the overall sample size, flagging did not appear to alter the variance of average whole‐brain GM thickness values (*var*_before_ = 0.0235; *var*_after_ = 0.0204; non‐significant reduction: *X* ^2^(1) = 0.84, *P* = 0.360), nor their correlation with age (*r*_before_(265) = −0.76, *P* < 0.001; *r*_after_(234) = −0.74, *P* < 0.001; non‐significant reduction: *z* = −0.43, *P* = 0.667). Critically, however a two‐sample Kolmogorov–Smirnov test confirmed that the vertex‐wise distribution of correlations between age and GM thickness exhibited a subtle but significant shift toward 0 after removing flagged participants (*D* = 0.0896, *P* < 0.001; see Fig. 7A). To illustrate the topography of regionally inflated effect sizes we measured the difference in correlation of age and GM thickness within the 148 anatomical parcels of FreeSurfer‐distributed Destrieux atlas [Destrieux et al., 2010] before versus after removing flagged scans. When removing flagged scans from the estimation sample, the effect sizes of age and GM thickness were attenuated across a majority of the cortex (86% of parcels), with the largest reductions along the cortical midline (e.g., precuneus, cingulate cortex, calcarine sulcus), bilaterally in the lateral temporal lobes, and in the right insula and right lateral parietal cortex (Fig. 7B,C).

## DISCUSSION

The present study revealed stable individual differences in head motion across functional scans, which were further correlated with QC ratings of T1w image quality. Moreover, head motion estimates from fMRI scans were shown to influence general estimates of individuals' brain structure, independent of the effects of age and rater‐defined quality. We adopted a procedure for “flagging” T1w scans based on combined measures of excessive fMRI head movement and low QC rating. Examining the flagged anatomical scans revealed that the regions most prone to potential movement‐related effects include many reported to undergo prominent atrophy with age. Taken together, the results suggest that natural in‐scanner head movements exert a potential confound on structural MRI measurements when left uncontrolled. This take home point is particularly relevant for studies comparing subgroups with high variability in head motion (e.g., older adults, adolescents, clinical populations), and/or studies examining multiple timepoints of data acquisition (i.e., longitudinal studies). In addition to describing the movement‐related observations we noted, we also offered readers some initial recommendations to overcome this potential source of bias in their own datasets.

A substantial body of research has focused on measuring and controlling for participant head motion during echo‐planar imaging [Friston et al., 1995; Jenkinson and Smith, 2001; Woods et al., 1992]. The nature of functional acquisitions (e.g., a BOLD image collected every TR) provides an opportunity to directly measure head position over the course of the scan. Since changes in head position are calculable frame‐to‐frame in EPI, a variety of methods have been developed to identify and account for motion‐related variance [e.g., frame censoring, regression; Jo et al., 2013; Patel et al., 2014; Power et al., 2014; Satterthwaite et al., 2013]. Likewise, the confound of motion artifacts remains an ongoing problem for structural brain imaging, where correction methods have not yet mitigated their bias. In contrast to echo‐planar imaging (e.g., fMRI, DWI), typical T1w structural acquisitions generate a single image over a several minute span without any direct estimations of how head position changes over the course of the scan. When direct measures have been available, one recent study has shown alarming inaccuracy in estimates of gray matter thickness and volume in the presence of head movements [Reuter et al., 2015], albeit in a smaller preliminary experiment that prescribed that motion.

Given that head motion confounds such measurement but is typically unavailable with T1w structural scans, we are met with a key challenge: how can we ensure that scans acquired without direct movement information are not biased by potential motion‐related artifacts? Although continued development of prospective methods will improve the way that future studies use structural imaging to study anatomy [see Zaitsev et al., 2015], development of such innovations is not applicable to a number of extremely valuable legacy datasets and to many other on‐going large‐scale data collection initiatives [e.g., ABIDE, ADHD‐200, ADNI, Betula, DLBS, FCON1000, HCP, HABS, NIMH adolescents, PNC, SLS; ADHD‐200‐Consortium, 2012; Biswal et al., 2010; Chan et al., 2014; Dagley et al., 2015; Di Martino et al., 2014; Giedd et al., 1999; Jack et al., 2008; Nilsson et al., 1997, 2004; Park et al., 2012; Satterthwaite et al., 2014; Schaie and Willis, 2010; Van Essen et al., 2012b, 2013]. While many studies have led efforts to correct the motion‐related bias in EPI, less work has demonstrated a suitable technique for mitigating the motion‐related bias on T1w imaging. To maximize data cleanliness, the sensitivity and reliability of morphometric findings, and predictive power/large sample size, a practical alternative is necessary to retroactively control for the motion‐related biases in T1w measures of brain anatomy.

One recommendation has been to assess image quality based on visual inspection, either removing problematic scans or reacquiring higher quality data when practical [Reuter et al., 2015]. However, a combination of intra‐ and inter‐rater variability and an under‐characterized framework for appraising MR image quality currently renders this approach sub‐optimal. We proposed an objective method to supplement QC ratings wherein independent scans that measure head motion in the same scan session (e.g., EPI sequences) are leveraged to identify potentially motion‐contaminated T1w images. Critically, QC ratings and $\bar{FD}$ predicted non‐overlapping variance in estimates of brain structure, suggesting that the two measures complement one‐another in flagging potentially problematic data points. Of note, T1w scans flagged by a combination of $\bar{FD}$ and QC ratings: (1) increased with increasing age, and (2) exhibited considerably reduced global and regional estimates of gray matter volume and thickness. Reductions in gray matter volume and thickness are well documented as a hallmark of healthy aging and cognitive decline [DeKosky and Scheff, 1990; Dickerson et al., 2008, 2012; Sowell et al., 2003]. We suggest that these effects may in some cases be overestimated, particularly in certain brain locations, by the inclusion of biased estimates from T1w structural scans with motion artifacts. The present work leveraged the variability in head motion and morphometry in a healthy adult sample to show that: (1) independent estimates of motion significantly predicted GM thickness (independent of age and gender), and (2) motion slightly but significantly biased thickness estimates in several regions that are often highlighted to undergo cortical thinning with increasing age [e.g., medial PFC, cingulate cortex, precuneus, IFG and anterior insula, SMG, lateral temporal cortex; Fjell et al., 2009; Lemaitre et al., 2012; Raz et al., 2005; Salat et al., 2004; Storsve et al., 2014].

It is important to note that many of the age‐associated differences in morphometry found with T1w imaging are robust neuroanatomical findings supported by more direct methods of anatomical measurement [e.g., histological studies of neuronal counts, cell density, and thickness; Morrison and Hof, 1997; Pakkenberg and Gundersen, 1997; Terry et al., 1987]. We emphasize that the removal of problematic T1w images in the present study did not negate the strong overall pattern of cortical thinning typically observed across the healthy adult lifespan, but instead highlight both where and how the effect size of age on thickness may be susceptible to misestimation when T1w scans with motion artifacts remain in a study sample. The present method of removing “flagged” scans from the estimation sample also reduced the association between head motion and GM thickness, suggesting that our flagging procedure may improve the accuracy of morphometric findings by mitigating the motion‐related bias. Looking forward, we suspect that when automated and semi‐automated methods of morphometry (e.g., ANTs, FSL, FreeSurfer) are used to compare populations where movement differences are more prominent or samples are smaller, the effects of head motion could be exacerbated and may incorrectly influence the conclusions drawn from the data [see Ducharme et al., 2016]. Moreover, it seems likely that motion‐related bias will limit other T1w image processing steps that rely on accurate brain anatomy; for instance, our preliminary observations suggest that T1w scans flagged according to ${\bar{FD}}_{all-task}$ and QC ratings exhibit reduced precision of within‐modality registration (see Supporting Information).

It is worth considering that fMRI motion here serves as a proxy measure for the motion that occurs during structural scans. Given that T1w scanning may non‐uniformly encode motion artifacts (e.g., movements during the middle of an MPRAGE scan can cause greater artifacts than motions near the start/end), more work is needed to evaluate how well fMRI motion estimates approximate the presence of movement‐induced artifacts in T1w images. In addition to mean FD values, researchers may consider using median or variance of motion to describe one's tendency to move during a scan session (e.g., to avoid bias from abnormal motion spikes). In the current sample, our findings were qualitatively unchanged when flagging with median FD (see Supporting Information). Also, since different fMRI motion algorithms may vary in precision [e.g., Ardekani et al., 2001; Morgan et al., 2001] they may differentially predict motion during T1w scans (e.g., SPM used here); notably, calculating outputs from another common motion algorithm (FSL's MCFLIRT) revealed high consistency of parameters ( ${\bar{FD}}_{all-task}$ between *r*(265) = 0.99, *P* < 0.001) and identified all but one of the scans flagged here. Another possibility is that motion estimates from subsets of scans that are temporally proximal and/or similar in task‐demands to the T1w acquisition are best suited for this prediction. For instance, the correlation matrix in Figure 2 suggests that the average movements in scans separated by greater intervening scans are nominally less correlated, particularly when participants exit the scanner between tasks (e.g., between “scenes3” and “rest” scans). While the current task‐ordered scanning protocol limited the interpretability of temporal and task effects, the highly significant pair‐wise relationships between individual rank ordering in $\bar{FD}$ across scans suggests that ${\bar{FD}}_{all-task}$ likely quantifies an accurate cross‐scan feature in individuals relative to a sample.

A recent study revealed a number of findings parallel to those reported here while examining fMRI motion and structural estimates in a large pediatric and young adult sample [Alexander‐Bloch et al., 2016]. Firstly, this previous report demonstrated that fMRI head motion may be stable across similar EPI scans. Second, the authors similarily identified a motion‐related bias in gray matter volume estimates of a stringently screened study sample (with visual QC). The present report not only confirms the prior findings, but also furthers these observations in important ways to demonstrate: (1) fMRI head motion might reflect a relatively stable within‐participant feature that persists across task demands and even over brief intervals where participants exit the scanner, (2) fMRI head motion and visual QC ratings exhibit statistically independent biases on multiple brain‐wide measures of morphometry derived with T1w images, and (3) flagging a relatively small number of T1w scans via a combination of fMRI head motion and QC ratings may significantly reduce the effect of motion‐related bias in analyses of morphometry. Notably, the significant continuous effect of fMRI motion parameters on T1w measures of whole‐brain and regional GM thickness observed in the current report was undetectable in the study be Alexander Bloch et al. [2016], possibly due to relatively less variability in head motion estimates than those found in the present adult lifespan dataset. Collectively, there is strong evidence that that average fMRI head motion measured within the same session is highly correlated within‐participant and may perform well as a proxy measure for motion‐related bias in scans without more direct measures of head movement. Additional work will be necessary to clarify precisely how motion‐related bias in T1w images varies and overlaps across these distinct study populations. Presently, we reiterate that future studies aiming to characterize accurately the morphometry of groups that differ in their tendency to move (e.g., younger vs. older adults, patients vs. controls) will be strengthened by considering whether their observed effects are robust to the motion‐related bias.

Though the discrepancy in $\bar{FD}$ and QC ratings as well as the similarity in head motion during EPI and T1w structural scans warrant further study, the distinct influences of $\bar{FD}$ and QC ratings reported here substantiate the consideration of both measures when analyzing structural MRI. The present findings indicate that the presence of biases in anatomical scans may be partially controlled by a combination of methods relying on rater‐defined QC and independent fMRI‐based estimates of head motion. It is important to point out that this strategy of “flagging” structural images with potential bias is distinct from that of correcting the image sequences themselves or using motion estimates as covariates in statistical analyses. Approaches that covary motion estimates may provide a reasonable strategy for controlling motion‐related biases on structural estimates; however, until ${\bar{FD}}_{all-task}$ values can be more closely related to motion‐induced artifacts in T1w scans it remains unclear whether using ${\bar{FD}}_{all-task}$ as a statistical covariate can accurately remove the motion confound in morphometric analyses. Improving T1w acquisition techniques will surely advance future studies of brain morphometry, however, improved QC and data screening may be the most practical alternative for existing datasets and ongoing data collection initiatives. Researchers must bear in mind that aggressive screening procedures can be inherently limited. For example, since high in‐scanner head motion and poor rater‐defined T1w quality can be strongly tied to variables of interest (e.g., aging, differences in diseased populations), flagging these data points for removal can result in sampling bias (i.e., only older adults or individuals healthy enough to stay still in the scanner will be studied). Notably, this inherent bias is not conceptually very different from the bias introduced by MRI eligibility requirements in many studies (e.g., no history of cardiovascular issues or head trauma) that already pre‐select a relatively healthy subpopulation. Experimenters need to be continually mindful in this trade‐off when adopting data screening techniques for T1w images.

An intriguing possibility is that dissimilarities in the propensity to move one's head during an imaging session is related to a trait‐like feature [e.g., impulsivity, Kong et al., 2014], and that this feature is a direct consequence of the local or global differences in brain anatomy highlighted here (e.g., Fig. 6). For instance, variation in the tendency to move during MRI might reflect a broader phenotype that is triggered in part by certain patterns of cortical thinning. One recent study reported that group‐based differences in the resting‐state correlations of the default mode network might distinguish high‐motion scans of high‐moving individuals from high‐motion scans of low‐moving individuals [Zeng et al., 2014]. While we cannot rule out a comparable scenario here, given that excessive motion has been shown to systematically bias structural measures in within‐subject longitudinal study designs [Reuter et al., 2015], we suspect that the reduced morphometric estimates found in the structural images flagged by the present report are a consequence of excessive movement during T1w scans rather than a cause. Still, additional work will be needed to characterize a potential link between gray matter morphometry and trait‐like head motion, particularly since a residual association between head motion and thickness persists after removing flagged scans; studying the extent to which head motion estimates are stable across multiple imaging sessions [see Reuter et al., 2015; Van Dijk et al., 2012; Zeng et al., 2014; Zuo et al., 2014] might help examine this possibility. To this end, it remains essential to relate the stability of movement patterns during fMRI to direct measures of head motion during T1w scanning. Nonetheless, given the nature of the noted biases we have reported, we maintain that within‐session head motion measurements (as used in this report) can provide a critical data‐flagging tool for removing potential motion‐related bias in structural imaging studies.

It is evident that motion‐related artifacts in structural MRI pose a potential limitation on measuring brain morphometry. Though most MRI research protocols acquire EPI images along with high‐resolution structural scans, if motion estimates from independent scans (e.g., fMRI, DWI) are also unavailable, investigators would have to rely exclusively on visual QC while accepting that some residual motion‐related bias is likely to limit their conclusions. Moreover, without adequate control for motion‐related bias, structural imaging studies requiring highly sensitive computational methods may be inherently limited (e.g., structural change in clinical trials). The present results are based on FreeSurfer estimates of morphometry, however the findings are likely to generalize to other structural estimation algorithms as well (e.g., ANTs, FSL, CIVET). As mentioned above, biased structural imaging may impair within‐ and between‐modality image registration, but also functional localization methods, and analyses that require accurate mapping to anatomical surfaces [McDonald et al., 2010; Wig et al., 2014], all of which often rely on accurate characterization of brain anatomy. Such problems with image registration may be exacerbated in cross‐cohort comparisons by warping T1w anatomical scans to a template image with varying degrees of success due to differences in motion‐related artifacts. The implication of this naturally extends to other measurements based on accurate estimates of brain anatomy including analysis of structural covariance networks [e.g., Zielinski et al., 2010; Montembeault et al., 2012], volume based morphometry [e.g., Schmitter et al., 2015], tract‐based spatial statistics [e.g., fractional anisotropy, diffusivity; Smith et al., 2006, 2007], and brain lesions imaged with FLAIR [e.g., white matter hyper‐intensities, infarctions; Hajnal et al., 1992; Brant‐Zawadzki et al., 1996], among many others.

## CONCLUSIONS

The present findings suggest that motion‐related bias in T1‐weighted structural MRI may be retrospectively flagged and removed, in part, via a combination of procedures relying on rater‐defined visual QC ratings and estimates of movement obtained from independent scans collected during the same scanning session. The inclusion of head motion estimates from other scans provides valuable information that would be missed by visual inspection alone. Broadly, the current findings may offer researchers a practical framework for objectively identifying problematic data points in other brain scans that also do not supply more direct measures of head motion (e.g., FLAIR, MRA). The observations highlighted warrant continued development and implementation of both qualitative and quantitative QC to improve the analysis of brain structure. The use of automated and semi‐automated methods in preparing and analyzing brain data are instrumental to enhancing the ways that researchers can examine functional and structural brain organization. Nonetheless, biased estimates of gray matter volume and thickness resulting from participant head motion during data collection is a serious issue that places important limits on the accuracy with which cross‐cohort anatomical differences and longitudinal change can be quantified. For this reason, we emphasize the importance of considering both visual inspection and objective motion‐related QC when assessing brain structure with MRI.

## Supporting information

Supporting Information
