# Supplementary material for: Motion‐related artifacts in structural brain images revealed with independent estimates of in‐scanner head motion
Source: Hum Brain Mapp. 2016 Sep 16;38(1):472–92. doi: 10.1002/hbm.23397 (PMC5217095; doi:10.1002/hbm.23397)
Supplement: Supplementary file 1 — Supporting Information [file HBM-38-472-s001.docx]

# Supplementary Material

# Motion-related artifacts in structural brain images revealed with independent estimates of in-scanner head motion

Neil K. Savalia^1^, Phillip F. Agres^1^, Micaela Y. Chan^1^, Eric J. Feczko^2^,

Kristen M. Kennedy^1^, & Gagan S. Wig^1,3^

^1^ Center for Vital Longevity and School of Behavioral and Brain Sciences, University of Texas at Dallas, Dallas, TX

^2^ Yerkes National Primate Research Center, Emory University, 201 Dowman Drive, Atlanta, GA

^3^ Department of Psychiatry, University of Texas Southwestern Medical Center, Dallas, TX

**SUPPLEMENTARY MATERIALS**

1. **SUPPLEMENTARY METHODS**
   1. Extended fMRI instructions
   2. Quality rating criteria for T1-weighted anatomical images (Table S1)
   3. Additional information on FreeSurfer editing procedures (Table S2)
2. **SUPPLEMENTARY RESULTS**
   1. Variation in the average frame-by-frame displacements of healthy adults is non-normal and related by heteroscedasticity across fMRI scans
   2. Cross-scan stability in head motion and biased thickness estimates are also revealed with median $\boldsymbol{FD}$ (Figure S1)
   3. Additional FreeSurfer-derived estimates of brain structure
      1. Independent effects of QC ratings and $\bar{\boldsymbol{FD}}$ on additional FreeSurfer-derived estimates of brain structure
      2. Motion-related bias in multiple measures of GM structure may be revealed in T1-weighted scans flagged by $\bar{\boldsymbol{FD}}$ and QC ratings
      3. GM volume estimates before and after removing flagged T1-weighted scans
   4. T1-weighted images flagged by $\bar{\boldsymbol{FD}}$ and QC ratings exhibit lower within-modality registration precision than demographically-similar control samples (Figure S2)
3. **REFERENCES**

# 1. SUPPLEMENTARY METHODS:

## 1.1. Extended fMRI Instructions

### The DLBS scanning session examined in the present report comprised seven fMRI scans consisting of three experimental tasks (i.e., ventral visual [‘VV’], ‘words’, and ‘scenes’) and a single ‘resting-state’ run (see Figure 1 and Table 1 of main manuscript). Visual stimuli were presented using E-prime software (Psychology Software Tools, Pittsburgh, PA, USA) projected through the back of the scanner and viewed with a mirror attached to the scanning head coil. For scans requiring active button pushes by the participant (i.e., words and scenes), responses were recorded using a fiber-optic button box held in the right hand. Participants first performed two runs of VV, then one run of words, three runs of scenes, and one run of resting-state. For each functional scan type, the experimental details along with the instructions given to each participant are presented below:

### *VV*: Participants completed two block-design runs of the VV task where they passively viewed gray-scale images from six item categories (e.g., human faces, primate faces, cat faces, houses, chairs, and phase-scrambled control images). Within each run, 24 pseudo-randomly arranged blocks of eight images (i.e., four blocks from each of the six categories) were presented successively such that each image appeared for two seconds with no inter-stimulus interval within a block. Images were taken primarily from the face library of Minear and Park ([Minear & Park, 2004](#_ENREF_2)), with certain animal photographs being taken from the Internet and control images being generated in-house. All images were presented only once. The experimenter verified that participants complied with the instructions and did not fall asleep during the functional scan via verbal confirmation. Each VV scan included a total of 202 volumes.

### *Words*: Participants completed a single run of a block-design semantic classification task. Participants viewed 128 nouns for 2.5 seconds each with a 0.5 second fixation crosshair between words. Words were presented in 16 blocks of 8 stimuli (24 seconds each) randomly interleaved with 3 rest blocks of fixation (24 seconds each). Participants made active (i.e., button-press) judgments on whether each noun referred a living or non-living item (half living, half non-living). Half of the items from each category were unambiguously living or nonliving (e.g., lion, radio), whereas the other half of the items were comparatively harder to classify (e.g., virus, zombie). All words were presented in lower-case white font in the center of a black background. Participants were presented with eight ‘easy’ blocks (eight words each), eight ‘hard’ blocks (eight words each), and three fixation blocks in a pseudo-random order. The words scan included a total of 231 volumes.

### *Scenes*: Participants completed three runs of an incidental encoding task where they viewed color images of outdoor landscape scenes and judged with a button press whether there was a body of water (e.g., lake, river, ocean) present in each image (see [Park et al., 2013](#_ENREF_3)). In each of the three runs, 32 images were presented in random order for three seconds each in an event-related design. A jittered inter-stimulus interval (consisting of a centrally presented crosshair) was used so that the time between stimuli ranged from 4-14 seconds. For each of the three scenes scans, a total of 171 volumes were collected. A surprise recognition memory task (consisting of previously seen images and foils) was administered to participants when they exited the scanner.

### *Resting-state*: Participants performed an eyes-open fixation resting-state scan. The participants were instructed to remain awake and fixated on a white crosshair presented in the middle of a black background. The experimenter verified that participants complied with the instructions and did not fall asleep during the functional scan via verbal confirmation. A single scan was collected with 154 volumes.

## 1.2. Quality rating criteria for T1-weighted anatomical images

### T1-weighted (T1w) images were rated for quality by qualified research assistants trained in FreeSurfer editing techniques. Raters derived visual QC score for participants’ original native T1w images (i.e., independent of FreeSurfer-processing) based on the qualitative severity of a combination of visually detectable scanner-related (e.g., signal inhomogeneity, radiofrequency distortions) and motion-related (e.g., ghosting, ringing) artifacts. Ratings were made, in part, based on data quality criteria available at: (<http://cbs.fas.harvard.edu/science/core-facilities/neuroimaging/information-investigators/qc>). For reference, a summary of the rating criteria we have compiled and used in the present study is listed in Table S1.

| **Artifact** | **Appearance** | **Cause** | **‘Pass’** | **‘Warn’** | **‘Fail’** |
| --- | --- | --- | --- | --- | --- |
| **Wrapping** | Scan field of view cuts off part of image with the missing piece appearing on the far side of image | Frequency reversal in image reconstruction incorrectly maps signal detected outside the MRI field of view to far side of image | Artifact is imperceptible or mildly detectable; brain anatomy data not affected by artifact | Artifact is present; non-brain tissue may overlap but does not severely impact brain anatomy itself | Substantial artifact detected; brain anatomy either cut off or overlapped by other anatomy |
| **Head Coverage** | Scan field of view cuts off part of image with the missing piece without reappearance elsewhere in image | Inadequate positioning of scanner frame of view when participant placed in MRI scanner | Head and brain are fully captured within the image frame of view | Incomplete head coverage; image may omit aspects of skull/non-brain tissue | Incomplete head and brain coverage; aspects of brain clipped from image |
| **Radiofrequency (RF) Noise** | Straight, rigid lines superimposed on anatomical image, or pronounced TV-static like noise | Electrical signal interference (e.g., static electricity) during image acquisition or imperfect electrical conduction in gradient coil | Artifacts not detected or faintly perceptible in default image visualization | Pronounced artifacts detected and may interfere with anatomy in focal brain locations over few image slices | Substantial RF spiking detected, widespread across brain in many image slices |
| **Susceptibility Artifact** | A non-anatomical black area/hole inside head, sometimes bordered by bright banding or ripples (e.g., ‘Moire Fringes’) | Often caused by metal in or near scanned area that shields tissue from scanner RF signal | Susceptibility is either not detected or present without interference with brain anatomy | Artifact present and possibly in close proximity to brain tissue, fringes/distortions may interfere with focal brain locations | Susceptibility has widespread presence or causes major signal loss in brain tissue |
| **Ringing, Striping, Blurring** | Light or dark circles, ripples, arcs in the image that often mirror the curve of features in the head/brain or signal smearing | Various; often caused by subject motion, but can also be attributed to MRI software/hardware malfunctioning | Artifacts not detected or faintly perceptible in default image visualization | Pronounced artifacts detected that may interfere with anatomy in focal brain locations over few image slices | Substantial and widespread artifacts, most image slices and brain locations impacted |
| **Shadowed Arc** | Light or dark arcs and circles that appear predominantly in white matter (e.g., centrum semiovale or internal capsule); similar to artifacts seen with ringing/striping/blurring | Image reconstruction of under-sampled brain data when using some parallel acquisition techniques | Artifacts not detected or faintly perceptible in default image visualization | Pronounced shadowing that may interfere with anatomy in focal brain locations or over few image slices | Substantial and widespread artifacts, most image slices and brain locations impacted |
| **Ghosting** | A faint and displaced copy of brain image or aspects of image (e.g., eyes, skull, brain); visually similar to artifacts seen with striping or blurring | Various; often caused by subject motion but also results from incorrectly mapped or shifted detection in signal channels | Artifacts not detected or faintly perceptible in default image visualization | Pronounced ghosting that may interfere with anatomy in focal brain locations or over few image slices | Substantial and widespread artifacts, most image slices and brain locations impacted |
| **Unexpected Signal Inhomogeneity** | Inconsistent or asymmetric signal intensity that renders image overly bright or overly dark in certain locations | Non-uniform signal sensitivity in scanner’s receiver coil array | Visibly uniform signal intensity within tissue-types throughout image | Inconsistent signal intensity present but consistent with coil profile, artifacts may impact focal brain location over few image slices | Inconsistent intensity profile is widespread, impacting most image slices and brain locations |

**Table S1.** Summary of T1-Weighted Artifacts and Quality Rating Criteria**.**

## 1.3. Additional information on FreeSurfer editing procedures

### Anatomical data was processed using FreeSurfer (v5.3). The processing pipeline requires careful inspection of automated processed outputs to assure that segmentations and reconstructions are spatially accurate and anatomically correct. At times, manual iterative intervention is required to correct errors. The editing process was based on instructions provided throughout the official FreeSurfer Wiki and editing tutorials (<http://freesurfer.net/fswiki/FreeSurferWiki>; <https://surfer.nmr.mgh.harvard.edu/fswiki/FreeSurferBeginnersGuide>). An in-house guide to our laboratory’s FreeSurfer editing procedure can be found at: <http://vitallongevity.utdallas.edu/cnl/>. An overview of the interventions used in correcting FreeSurfer data is presented in Table S2.

### Briefly, trained researchers check each participant’s FreeSurfer output (from ‘reconall -all’) visually by overlaying white and pial surface reconstructions on native anatomical images. If automated errors are detected (see Table S2), the researcher performs manual edits on T1-derived volumetric images to correct the defects; researchers carefully record the type of edits required (see Table S2) and specific placement of edits on a centralized FreeSurfer-checking database. Once the edits for a single participant are completed, the participant’s surfaces are reconstructed based on the edited inputs (i.e., using ‘autorecon’ options of ‘recon-all’). The researcher then re-checks the participant’s data to confirm whether the defects originally present are resolved based on the edits they have put in place. If inaccuracies persist after a single round of manual edits, or if new errors arise, the researcher will perform another iteration of edits and reconstruct the participant’s FreeSurfer data. The researcher continues re-checking and re-running cortical reconstruction until they determine that errors have been resolved. Finally, an independent researcher visually checks the participant’s FreeSurfer outputs and verifies them for anatomical accuracy. This two-rater process of verification helps ensure the editing process is thorough and adequate. The editing procedure tends to reduce the average thickness values measured by FreeSurfer (paired *t*-test: *t*(265) = 13.47, *p* < .001); critically the reductions are not statistically different for participants flagged by QC ratings and ${\bar{\boldsymbol{FD}}}_{\boldsymbol{all}\mathbf{-}\boldsymbol{task}}$ relative to the retained participants (*F*(1, 264) = 2.06, *p* = .152). Importantly, editing did not differentially impact flagged samples.

| Editing Tool | Error Appearance | Editing Technique | Result of Intervention |
| --- | --- | --- | --- |
| Control points | Inappropriate intensity normalization results in erroneous white matter segmentation and placement of white matter surfaces (i.e., at the grey-matter-white-matter boundary) and/or pial surfaces (i.e., at the grey-matter-pia-mater boundary) such that locations belonging within the segments/boundaries are excluded. | Locations that are determined to reside within the appropriate boundary but are missed by the white matter mask or white/pial surfaces are labeled with a control point. User-defined extensions of white matter segments and/or white/pial boundaries are introduced by placing a track of control points separated by several voxels and image slices in three dimensions. | Newly generated white matter segmentations and placement of white/pial surfaces are based on an iteration of intensity normalization that takes user-defined control points into account. Newly generated outputs include locations that were previously excluded from either the white matter mask or white surfaces. |
| White matter edits | Inappropriate segmentation of white matter or inadequate generation of white surface boundaries (at the grey-matter-white-matter boundary) either erroneously includes (e.g., blood vessels, optic tract) or excludes (e.g., lateral ventricles, white matter hypo-intensities in T1w image) focal brain locations. | Locations that are erroneously included in the white matter segmentation or white surfaces are manually removed from the FreeSurfer-generated white matter mask. Conversely, erroneously excluded white matter locations are filled back into the white matter mask. | Regenerated white matter segmentations and placement of white matter surfaces are based on an iteration of intensity normalization that utilizes the manually edited white matter mask. Newly generated outputs both include locations that were previously excluded or exclude regions that were previously included. |
| Pial edits | Errors associated with inadequate skull-stripping procedures can result in an inappropriate grey matter ribbon or pial surfaces that are placed incorrectly along the brain’s cerebral mantle. | Locations where an under-aggressive skull-stripping procedure results in pial surfaces extending off the cerebral mantle (e.g., dura, cerebellum) will be removed; grey matter locations excluded from the pial surface/grey matter ribbon by an over-aggressive skull-stripping are recovered from the native T1w image. | Regenerated outputs (e.g., grey matter ribbon and labels, pial surfaces) are more accurately placed along the grey-matter-pia-mater boundary. Newly generated segments and surfaces are contained by the user-defined edits to the refined skull-stripped image. |

#### Table S2. Summary of FreeSurfer Editing Procedures

# 2. SUPPLEMENTARY RESULTS:

## 2.1. Variation in the average frame-by-frame displacements of healthy adults is non-normal and related by heteroscedasticity across fMRI scans

### The variation in $\bar{\boldsymbol{FD}}$ (e.g., Figure 5C of main report) across healthy adults is heavily concentrated around a comparatively low sample mean (i.e., high peakedness or kurtosis) with several individuals with high $\bar{\boldsymbol{FD}}$ that result in a distribution that is skewed to the right. Comparisons of statistically associated probability distributions that are overly kurtotic and/or skewed (i.e., do not follow a normal distribution) may result in heteroscedasticity, a situation where the variability in one measure is non-uniformly predicted by another measure. Such a scenario can be challenging for inference between variables because the error associated with predicting one measure from the other is also non-uniform. A primary objective of this report was to quantify how head displacements from one fMRI scan could be used to infer motion in a separate scan, even if such relationships were heteroscedastic. As such, we tested whether the variation in $\bar{\boldsymbol{FD}}$ from individual scans statistically diverged from a normal distribution and whether their interrelationships were significantly heteroscedastic.

### First, we used the Shapiro-Wilk test ([Shapiro & Wilk, 1965](#_ENREF_5); [Royston, 1982](#_ENREF_4)) to examine whether the dispersion in measured $\bar{\boldsymbol{FD}}$ from the seven fMRI scans of this study differed from a normal distribution. All seven distributions were significantly non-normal (all *p*s < .001 after Bonferroni correction for 7 simultaneous comparisons). Next, we used the White test ([White, 1980](#_ENREF_6)) to assess the presence of heteroscedasticity in the regression of each scan’s distribution of $\bar{\boldsymbol{FD}}$ to that of every other scan (42 total tests). A total of 36 of the 42 tests indicated the presence of significant heteroscedasticity after Bonferroni correction for the 42 simultaneous tests. Given that the measures of $\bar{\boldsymbol{FD}}$ from all seven scans were non-normally distributed and the majority of $\bar{\boldsymbol{FD}}$-$\bar{\boldsymbol{FD}}$ relationships were characterized by significant heteroscedasticity, a strictly linear dependence between the head motions of different scans could not be assumed. Accordingly, we opted to characterize the relationships between different run $\bar{\boldsymbol{FD}}$ values using Spearman’s rank correlation coefficients, which measure the association between the rank-order of two variables (and is thus immune to the heteroscedasticity of variables themselves), are reported in the main document of this report (see Figure 2A).

### Of importance, there was no statistical evidence for heteroscedasticity in the relationship of age and $\bar{\boldsymbol{FD}}$, or for any of the relationships of anatomical estimates and $\bar{\boldsymbol{FD}}$ (all *p*s > .15). Thus, the linear dependence of $\bar{\boldsymbol{FD}}$ on age or anatomy was characterized with Pearson’s product moment correlation coefficients throughout the main report.

## 2.2. Cross-scan stability in head motion and biased thickness estimates are also revealed with median $\boldsymbol{FD}$

Since in-scanner motion may potentially manifest as abrupt head movements (e.g., head twitch, position adjustment) separated by periods of stability, median motion may provide a more accurate representation of the central tendency (less susceptible to outliers) of individuals’ frame-by-frame movements than mean measures (${\bar{\boldsymbol{FD}}}_{\boldsymbol{all}\mathbf{-}\boldsymbol{task}}$). We replicated the primary analyses of main report using median $\boldsymbol{FD}_{\boldsymbol{all-task}}$ (median of the median $FD$ for each task). Since mean ${\bar{\boldsymbol{FD}}}_{\boldsymbol{all}\mathbf{-}\boldsymbol{task}}$ and median $\boldsymbol{FD}_{\boldsymbol{all-task}}$ are highly correlated (Spearman’s *r*(265) = .97, *p* < .001) and overlap in flagging all but one of the same group of participants, we predicted that the median estimator would generally confirm the findings of the main report. The replication results using median $\boldsymbol{FD}$ were qualitatively very similar to the results presented in the main report:

1. Median $\boldsymbol{FD}$ is correlated within individuals across fMRI scans.

Within individuals in our dataset, the median $\boldsymbol{FD}$ for each fMRI scan is highly correlated across all other functional runs (all *p*s < .001 after Bonferroni correction for 21 comparisons; correlation measured with Spearman’s correlation; Figure S1A).

1. QC ratings covary with median $\boldsymbol{FD}_{\boldsymbol{all-task}}$ independent of age.

We computed the ANCOVA of median $\boldsymbol{FD}_{\boldsymbol{all-task}}$ (dependent measure) including QC rating as a between-subject factor and participant age as a continuous predictor (*F*(5, 260) = 19.69, *p* < .001, adj. *R^2^* = .261). Lower QC ratings predicted increasing median $\boldsymbol{FD}_{\boldsymbol{all-task}}$ (*F*(2, 260) = 19.23, *p* < .001) independent of a main effect of aging on increasing median $\boldsymbol{FD}_{\boldsymbol{all-task}}$ (*F*(1, 260) = 20.68, *p* < .001). The interaction of QC ratings and aging on median $\boldsymbol{FD}_{\boldsymbol{all-task}}$ was not significant (*F*(2, 260) = 1.16, *p* < .314).

1. Median $\boldsymbol{FD}_{\boldsymbol{all-task}}$ and QC ratings predict independent variance in GM thickness estimates, independent to the effects of age.


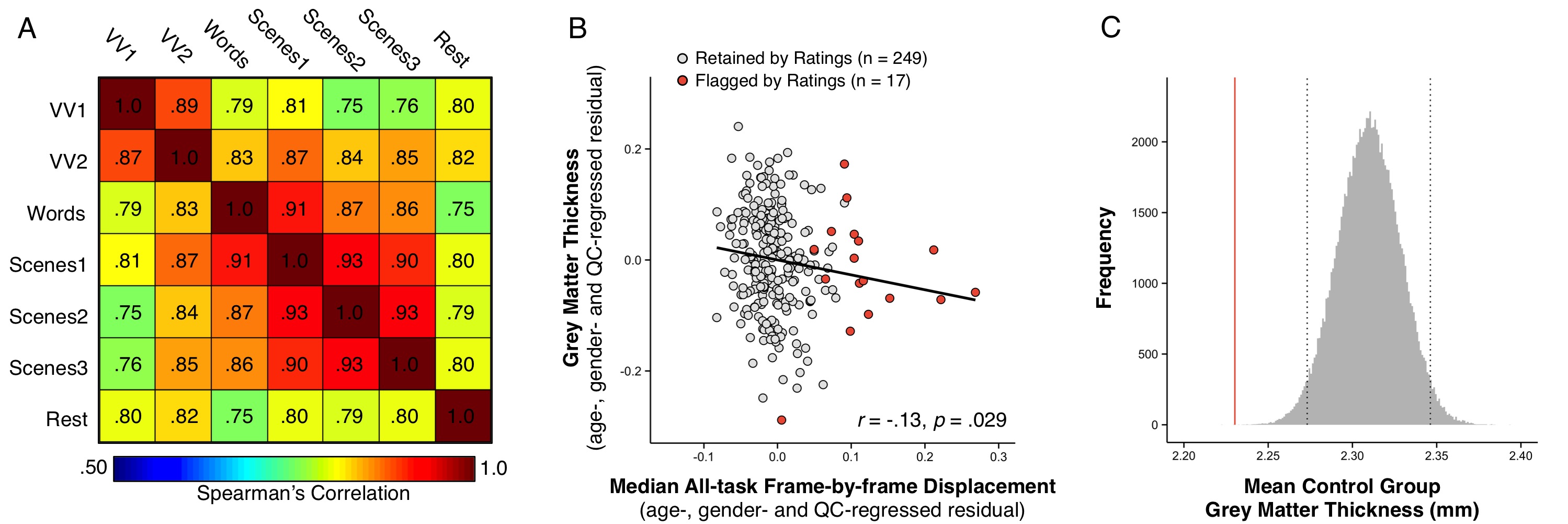
We computed the ANCOVA using median $\boldsymbol{FD}_{\boldsymbol{all-task}}$ (continuous predictor), QC ratings (between-subject factor), age (continuous predictor), and gender (nuisance covariate) as predictors for average whole-brain grey matter thickness (dependent measure). The model fit was significant (*F*(23, 242) = 20.91, *p* < .001, adj. *R^2^* = .633), and no interactions were found to be significant (all *p*s > .474). We found a significant main effect of increasing age on decreased thickness estimates (*F*(1, 242) = 220.30, *p* < .001) and a significant main effect of lower visual QC ratings on reduced GM thickness (*F*(2, 242) = 11.89, *p* < .001). There was a marginal main effect of increasing median $\boldsymbol{FD}_{\boldsymbol{all-task}}$ on reduced thickness estimates (*F*(1, 242) = 2.94, *p* = .088). Furthermore, there was a significant partial correlation of median $\boldsymbol{FD}_{\boldsymbol{all-task}}$ and GM thickness after removing the effects of age, gender and QC ratings on both variables (*r*(264) = -.13, *p* = .029; Figure S1B); in parallel, there was a significant partial correlation of QC ratings and GM thickness after removing the effects of age and median $\boldsymbol{FD}_{\boldsymbol{all-task}}$ on both variables (*r*(264) = .29; *p* < .001).

**Figure S1. Median** $\boldsymbol{FD}$ **results in similar observations as shown with** $\bar{FD}$**.** (a) Participants’ median $FD$ is significantly correlated across all fMRI scans (Spearman’s correlation, all *p*s < .001 after Bonferroni correction). (b) Increasing median $\boldsymbol{FD}_{\boldsymbol{all-task}}$is significantly associated with reductions in grey matter thickness, independent of age, QC ratings and gender (i.e., partial correlation). (c) Participants flagged via a combination of QC rating ‘fails’ and median $\boldsymbol{FD}_{\boldsymbol{all-task}}$ 1.5SD above the sample mean (total n = 30) exhibit significantly lower mean thickness estimates (vertical red line) than 100,000 age- and gender-matched bootstrapped control groups (*p* = .00002).

1. Participants flagged with elevated median $\boldsymbol{FD}_{\boldsymbol{all-task}}$ and poor QC ratings have systematically reduced GM thickness estimates.

A total of 30 individuals’ T1w scans were flagged for containing potentially motion-related bias (i.e., total combining from both rating ‘fails’ and median $\boldsymbol{FD}_{\boldsymbol{all-task}}$ 1.5SD above the sample mean). A bootstrap procedure was used to create gender- and age-matched control samples for this motion-contaminated group (see main report Methods). The average GM thickness of the flagged group was significantly reduced relative to control samples (*p* = .00002; Figure S1C).

The central findings of this report are largely unchanged when using median $\boldsymbol{FD}_{\boldsymbol{all-task}}$ in place of ${\bar{\boldsymbol{FD}}}_{\boldsymbol{all}\mathbf{-}\boldsymbol{task}}$, and both measures appear to predict unique variance in grey matter thickness estimates that is unrelated to QC ratings and age. Similar relationships may be likely when using other indices of fMRI motion to predict morphometric measures derived from structural scans that harbor motion-related bias. We hypothesize that both patterns of motion are important for biasing structural estimates. Although the median estimator may be advantageous for identifying individuals who consistently move their head over a large displacement at every frame (e.g., 0.40 mm displacement every frame) as high movers, it will simultaneously be less sensitive to identifying individuals who habitually exhibit sharp motion spikes in less than 50% of their EPI frames (e.g., 1 mm displacement every third frame, .10 mm displacement all other frames). Given this trade-off, we have chosen to continue using the mean (${\bar{\boldsymbol{FD}}}_{\boldsymbol{all}\mathbf{-}\boldsymbol{task}}$) as our measure of interest in the main report, though it remains to be seen whether median or other independent estimates of head motion (e.g., variance-based) best predict motion-related bias in T1w images.

## 2.3. Effects of QC ratings and $\bar{\boldsymbol{FD}}$ on additional FreeSurfer-derived estimates of brain structure

### The primary focus of the present report was to explore motion-related biases on estimates of grey matter thickness. However, additional FreeSurfer morphometric statistics were quantified and could be related to independent estimates of motion-related bias.

### 2.3.1. Independent effects of QC ratings and $\bar{\boldsymbol{FD}}$ on additional FreeSurfer-derived estimates of brain structure

### We examined the relationship between additional FreeSurfer morphometric estimates and user-defined T1w quality and ${\bar{\boldsymbol{FD}}}_{\boldsymbol{all}\mathbf{-}\boldsymbol{task}}$ using the general linear model. Three ANCOVA models were constructed with either cortical GM volume, subcortical GM volume, and GM surface area as dependent variables and using QC ratings, ${\bar{\boldsymbol{FD}}}_{\boldsymbol{all}\mathbf{-}\boldsymbol{task}}$, and age as predictors with intracranial volume included as a covariate. All model fits were significant (all *p*s < .001, all adj. *R*^2^ > .391) and included significant main effects of increasing age on decreasing structural estimates (all *p*s < .001). In addition, decreasing cortical grey matter volume was predicted independently by increasing ${\bar{\boldsymbol{FD}}}_{\boldsymbol{all}\mathbf{-}\boldsymbol{task}}$ (*F*(1, 242) = 3.37, *p* = .003) and lower QC ratings (*F*(2, 242) = 2.02, *p* = .045, reduced subcortical grey matter volume was predicted by ${\bar{\boldsymbol{FD}}}_{\boldsymbol{all}\mathbf{-}\boldsymbol{task}}$ (*F*(2, 242) = 2.44, *p* = .026) but not QC ratings (*F*(2, 242) = 1.39, *p* = .201), and reduced total cortical surface area were predicted marginally by increasing ${\bar{\boldsymbol{FD}}}_{\boldsymbol{all}\mathbf{-}\boldsymbol{task}}$ (*F*(1, 242) = 2.08, *p* = .056) but not QC ratings (*F*(2, 242) = 0.70, *p* = .691). Based on the above we note that potential motion-related reductions in grey matter volume and surface area might be better predicted by increased ${\bar{\boldsymbol{FD}}}_{\boldsymbol{all}\mathbf{-}\boldsymbol{task}}$ than by T1w image quality ratings. This also reinforces our suggestion that each variable ($\bar{\boldsymbol{FD}}$ and QC rating) may contribute non-overlapping sources of important information for data cleanliness.

### 2.3.2. Motion-related bias in multiple measures of GM structure may be revealed in T1-weighted scans flagged by $\bar{\boldsymbol{FD}}$ and QC ratings

### As reported above in 2.6.1, subcortical and total grey matter volumes and total cortical surface area were sensitive to ${\bar{\boldsymbol{FD}}}_{\boldsymbol{all}\mathbf{-}\boldsymbol{task}}$ and QC ratings. Importantly, it remained possible that motion-contaminated sample (n = 31) flagged by both quality ‘fails’ and elevated ${\bar{\boldsymbol{FD}}}_{\boldsymbol{all}\mathbf{-}\boldsymbol{task}}$ (i.e., screened 1.5SD > sample mean) significantly differed from matched control groups in terms of these volumetric estimates. As described in the main report, a bootstrap analysis was performed to randomly reselect 100,000 control samples that were age and gender matched to the 31 flagged individuals. While subcortical grey matter volume (*p* = .186) and total grey matter surface area (*p* = .384) did not significantly differ between flagged and control groups, total grey matter volume was found to be reliably reduced in the motion-contaminated (flagged) sample relative to matched controls (*p* = .001). Parallel to observations with grey matter thickness (see main Results), total grey matter volume exhibited a nominally stronger correlation with age before removing flagged scans (*r*(265) = -.670, *p* < .001) than after (*r*(234) = -.653, *p* < .001) although change in correlation was not significantly different (*z* = -.33, *p* = .741). While it remains possible that regional effect sizes of GM volume and age are altered by the flagging procedure, a finer-grained analysis was outside the scope of the current paper.

## 2.4. T1-weighted images flagged by $\bar{\boldsymbol{FD}}$ and QC ratings exhibit lower within-modality registration precision than demographically-similar control samples

## While the main report confirms that T1w scans flagged for motion-related bias exhibit significantly reduced automated estimates of morphometry (Main Results 3.4), it remains important to determine whether other aspects of data processing and analysis are impacted when conducted with T1w scans flagged by $\bar{\boldsymbol{FD}}$ and QC ratings. For instance, a crucial step in many neuroimaging studies is to perform both intra- and inter-individual image registration both within and across imaging modalities (e.g., alignment of BOLD fMRI images from one participant to their T1w structural image, alignment of many participants’ T1w structural scans to a single sample-based brain atlas). Accurate registration improves individual and group-based localization of imaged effects, allowing researchers to draw stronger statistical inferences about brain structure and function. However, it is possible that movement-related T1w artifacts obscure the representation of actual brain features (e.g., contrast along tissue-type boundaries) that are used to guide the alignment of T1w structural images to a target image. As such, we hypothesized that image registration of T1w images to a group-based anatomical brain atlas would be compromised for participants flagged by $\bar{\boldsymbol{FD}}$ and QC ratings.

## The automated FreeSurfer processing pipeline involves calculating an affine transformation (12 degrees of freedom) to align individuals’ T1w scans to the MNI305 atlas using the 4dfp software suite. The image similarity metric *η^2^* (eta-squared; e.g., see [Cohen et al., 2008](#_ENREF_1)) was used to measure the precision of alignment of between each participant’s T1w image and the MNI305 atlas. FreeSurfer calculates *η* (eta) between T1w scans and the MNI305 atlas as part of the default processing pipeline, which can in turn be extracted from FS log files (e.g., ‘grep “TalAviQA:” recon-all.log’). As in the Main Results 3.4, the average *η^2^* of the 31 participants flagged by $\bar{\boldsymbol{FD}}$ and QC ratings was compared against the average *η^2^* measured for 100,000 age- and gender-matched control samples (Figure S5; see main report for methodological details on bootstrapping procedure). Critically, the T1w scans of flagged participants had significantly reduced *η^2^* similarity to the atlas target compared to bootstrapped control groups (*p* = .00022).

## As predicted, T1w scans flagged using $\bar{\boldsymbol{FD}}$ and QC ratings exhibited a significant bias (reduction) in registration precision relative to matched controls. In combination with the finding that these flagged images also result in reduced estimates of FS-based morphometry, our results suggest that the ability to use flagged T1w scans may be compromised across multiple processing operations. Additional work is needed to determine how motion-related differences in T1w registration precision relate to the statistical relationships found using T1w images or other scans that are registered based on T1w images (e.g., fMRI).

**Figure S2. Participants flagged by** $\bar{\boldsymbol{FD}}$ **and QC ratings are less accurately registered to the MNI305 atlas.** The 31 participants flagged for motion-related bias exhibited biased registration accuracy to the MNI305 atlas compared to 100,000 age- and gender-matched bootstrapped control groups (*p* = .00022): (a) a bar plot depicting the significant reduction in registration similarity (*η^2^*) for flagged participants relative to demographically-similar control groups of the same sample size (error bar is 95% confidence interval around mean from all bootstraps); (b) a histogram depicts the distribution of mean *η^2^* for all bootstrapped control samples with a vertical orange line representing the significantly reduced average *η^2^* measured for the 31 flagged participants.


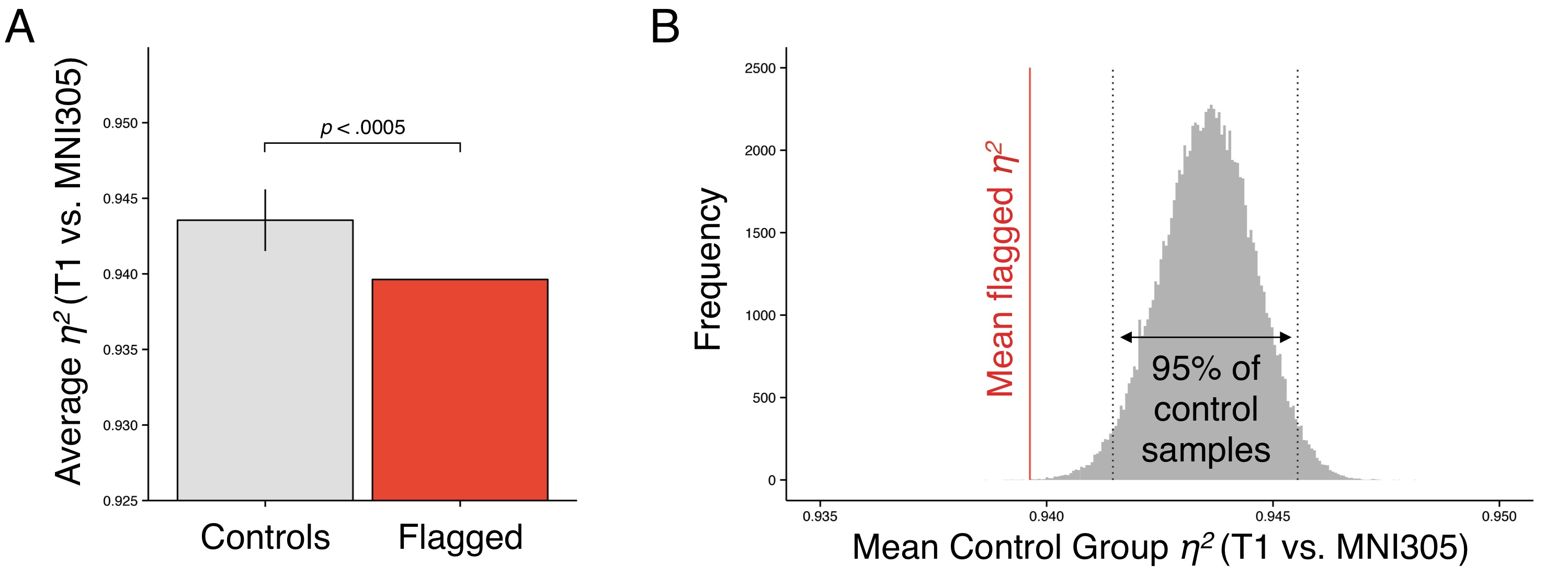


## 3. REFERENCES

### Cohen, A. L., Fair, D. A., Dosenbach, N. U., Miezin, F. M., Dierker, D., Van Essen, D. C., Schlaggar, B. L., & Petersen, S. E. (2008). Defining functional areas in individual human brains using resting functional connectivity MRI. *Neuroimage, 41*(1), 45-57. doi: 10.1016/j.neuroimage.2008.01.066

### Minear, M., & Park, D. C. (2004). A lifespan database of adult facial stimuli. *Behav Res Methods Instrum Comput, 36*(4), 630-633.

### Park, H., Kennedy, K. M., Rodrigue, K. M., Hebrank, A., & Park, D. C. (2013). An fMRI study of episodic encoding across the lifespan: changes in subsequent memory effects are evident by middle-age. *Neuropsychologia, 51*(3), 448-456. doi: 10.1016/j.neuropsychologia.2012.11.025

### Royston, J. P. (1982). An Extension of Shapiro and Wilk-W Test for Normality to Large Samples. *Applied Statistics-Journal of the Royal Statistical Society Series C, 31*(2), 115-124. doi: Doi 10.2307/2347973

### Shapiro, S. S., & Wilk, M. B. (1965). An Analysis of Variance Test for Normality (Complete Samples). *Biometrika, 52*, 591-&. doi: Doi 10.2307/2333709

### White, H. (1980). A Heteroskedasticity-Consistent Covariance-Matrix Estimator and a Direct Test for Heteroskedasticity. *Econometrica, 48*(4), 817-838. doi: Doi 10.2307/1912934
